# Patient-specific CFD modeling of CSF flow in Chiari I malformation: denticulate-ligament-induced compartmentalization explains flow patterns

**DOI:** 10.1186/s12987-026-00780-y

**Published:** 2026-03-02

**Authors:** Guillermo L. Nozaleda, Francisco J. Parras-Martos, Carolyna Yamamoto, Wilfried Coenen, Carlos Martínez-Bazán, Gonzalo Olivares-Granados, Nicolás Cordero-Tous, Vijay M. Ravindra, Cándido Gutiérrez-Montes, Antonio L. Sánchez

**Affiliations:** 1 Department of Mechanical and Aerospace Engineering, University of California San Diego, La Jolla, CA 92093-0411 USA; 2 Área de Mecánica de Fluidos, Departamento de Ingeniería Mecánica y Minera, Universidad de Jaén, Jaén, 23071 Spain; 3 Andalusian Institute for Earth System Research, Universidad de Jaén, Campus Las Lagunillas s/n, Jaén, 23071 Spain; 4 Department of Biomedical Engineering, Johns Hopkins University School of Medicine, Baltimore, MD 21218 USA; 5 Grupo de Mecánica de Fluidos, Departamento de Ingeniería Térmica y de Fluidos, Universidad Carlos III de Madrid, Leganés, 28911 Spain; 6 Área de Mecánica de Fluidos, Departamento de Mecánica de Estructuras e Ingeniería Hidráulica, Universidad de Granada, Granada, 18071 Spain; 7 Andalusian Institute for Earth System Research, Universidad de Granada, Avenida del Mediterráneo s/n, Granada, 18006 Spain; 8 Unidad de Neurocirugía Funcional, Departamento de Neurocirugía, Hospital Universitario Virgen de las Nieves, Granada, 18014 Spain; 9 Departamento de Anatomía y Embriología Humana, Facultad de Medicina, Instituto de Investigación Biosanitaria ibs. GRANADA, Universidad de Granada, Granada, 18016 Spain; 10 Departamento de Cirugía y sus Especialidades, Facultad de Medicina, Universidad de Granada, Granada, 18016 Spain; 11 Division of Pediatric Neurosurgery, Primary Children’s Hospital, Salt Lake City, UT 84113 USA; 12 Department of Neurosurgery, University of Utah School of Medicine, Salt Lake City, UT 84113 USA

**Keywords:** CSF flow, CFD modeling, Denticulate ligaments, Chiari I malformation

## Abstract

Computational fluid dynamics (CFD) has been widely used to study cerebrospinal fluid (CSF) flow in Chiari Malformation Type I (CM-I). However, most approaches rely on limited patient-specific detail, and it remains unclear whether such minimal input is sufficient to yield physiologically realistic flow predictions. In this study, we construct a series of MRI-based models of the craniocervical CSF space in a CM-I patient, complemented with representations of microanatomical features derived from ex vivo measurements and patient MRI data, to assess how CFD predictions are influenced by the choice of boundary conditions in the numerical integrations and the inclusion or omission of nerve roots and denticulate ligaments in the anatomical model. Our results reveal that while increasing patient-specific detail in boundary conditions improves agreement with velocity fields measured via phase-contrast MRI, key flow features—most notably anterior–posterior compartmentalization and bidirectional patterns during flow reversal—only emerge when denticulate ligaments are included in the model. In contrast, inclusion of nerve roots has a more localized effect on the velocity field and a modest impact on pressure drops. Our findings not only clarify how more detailed boundary conditions and improved anatomical fidelity affect velocity and pressure predictions, but also provide a mechanistic explanation for flow patterns commonly observed in CM-I that have remained unexplained, highlighting the critical role of denticulate ligaments.

**Supplementary Information:**

The online version contains supplementary material available at 10.1186/s12987-026-00780-y.

## Background

Chiari Malformation Type I (CM-I) is a neurological disorder in which the cerebellar tonsils (the lower part of the cerebellum) herniate into the upper spinal canal. This downward displacement can partially or completely obstruct the cerebrospinal fluid (CSF) space at the craniocervical junction  [1, 2], disrupting the normal oscillatory CSF flow driven by cardiac and respiratory cycles  [3]. CM-I is often associated with symptoms such as headaches, neck pain, dizziness, and balance disturbances. Understanding the hydrodynamic aspects of CM-I is critical, as altered CSF dynamics are believed to be connected with symptom severity  [4] and the development of associated conditions such as hydrocephalus  [5] or syringomyelia  [6].

Advances in magnetic resonance imaging (MRI) have been instrumental in deepening our understanding of CSF dynamics in CM-I  [4]. In particular, phase-contrast MRI (PC-MRI) offers a noninvasive assessment of flow abnormalities in these patients. Previous PC-MRI studies have shown that CM-I is associated with abnormal peak velocities, with most reporting elevated peak velocities relative to healthy subjects  [2, 7–12], while a few have reported reduced peak velocities  [13, 14]. CM-I is also marked by increased spatial variability of the flow, with patients frequently showing high-velocity jets bilaterally in the anterior subarachnoid space and minimal or absent flow posteriorly  [9–11, 15], which contrasts with the more uniform distributions observed in healthy individuals. These flow abnormalities tend to normalize following posterior fossa decompression  [16–19], which remains the standard treatment for CM-I.

While PC-MRI alone is a powerful diagnostic tool for CSF dynamics, its combination with computational fluid dynamics (CFD) offers additional advantages. First, CFD allows for detailed analysis of many aspects of the flow dynamics  [20–24], including noninvasive estimation of pressure gradients—information otherwise inaccessible without lumbar puncture  [25]. Second, whereas PC-MRI typically provides cardiac-gated velocity measurements at discrete locations, and along a single direction (e.g., perpendicular to the imaging plane in 2D PC-MRI), CFD allows for the full reconstruction of volumetric, time-resolved velocity fields. The latter reduces the need for time-consuming acquisitions such as 4D flow MRI (time-resolved 3D PC with three-directional velocity encoding  [26]), which are often unavailable in standard clinical settings. Access to this information through CFD simulations has enabled, for instance, the assessment of coughing effects on CSF flow dynamics  [27], as well as the evaluation of hydrodynamic markers in CM-I studies, beyond purely geometrical or velocity-based parameters  [2, 28, 29]. Notable examples of such markers include maximum spatial pressure variations  [2, 28, 30] and longitudinal impedance  [31–33] (a frequency-dependent metric relating oscillatory pressure and flow along the spinal canal), both of which have been shown to correlate with symptom severity.

Most CFD studies on CM-I incorporate patient-specific information, but this is often limited to geometrical reconstructions from MRI data, with generic boundary conditions applied in the absence of subject-specific measurements  [33, 34]. This is common in retrospective analyses, where velocity data are typically unavailable. More personalized approaches incorporate patient-specific velocity measurements to define boundary conditions, but even then, they typically assume spatially uniform inlet velocities and zero pressure at the outlet  [28, 30–32, 35]—simplifications that may not accurately reflect physiological conditions and whose generalized use is partially driven by their ease of implementation in commercial CFD solvers. Støverud et al.  [36] advanced this by prescribing spatially varying inlet velocity profiles, though these were resolved in space using a wall-based weighting function rather than from PC-MRI–derived spatial distributions. Additionally, most CFD models are anatomically simplified, omitting small but potentially influential structures such as nerve roots and denticulate ligaments, whose effects remain largely unexplored in CM-I. An exception is the study by Pahlavian et al.  [37], who constructed CFD models of the cervical spine including idealized nerve roots and ligaments, for both a healthy subject and a CM-I patient. However, they still assumed a spatially uniform inlet velocity and zero pressure at the outlet, thereby potentially limiting the physiological fidelity of their simulations. A further limitation of many prior studies is the lack of comprehensive validation of CFD predictions against MRI velocity measurements. A notable exception is the work of Pahlavian et al.  [22], who used data from a healthy volunteer to construct an in vitro model of the craniocervical SAS and compare 4D flow MRI measurements with CFD predictions. Their analysis provided important insights into the accuracy of CFD predictions relative to 4D flow MRI, including the need to spatially average CFD results prior to comparison with MRI data. However, the study was limited to data from a healthy case and did not include denticulate ligaments into the model. Altogether, these limitations motivate the development of more physiologically realistic computational models in anatomically detailed geometries, combined with systematic validation against in vivo MRI measurements.

In this study, we assess various CFD models to examine the implications of common modeling assumptions. Geometrical reconstructions are derived from T2-weighted MRI, while boundary conditions are informed by cardiac-gated 2D PC-MRI velocity measurements, with additional measurements used for model validation. Beyond assessing the influence of boundary conditions on flow predictions, we investigate the role of nerve roots and ligaments, with the latter shown to be fundamental in shaping the CSF flow patterns observed in CM-I.

## Methods

### Magnetic resonance imaging

A male patient (51 years old, 79 kg) diagnosed with CM-I and syringomyelia participated in this study. MRI scanning was performed at the CIMCYC research center (Granada, Spain). All experimental protocols were approved by the institutional review board, and all procedures were conducted in accordance with relevant guidelines and regulations. Written informed consent was obtained from the participant prior to enrollment in the study.

Imaging was performed on a 3T Magnetom Prisma Fit MRI scanner (Siemens) using a 64-channel head and neck coil in combination with a 32-channel spine coil. High-resolution images of the head and spine were acquired using a 3D T2-weighted sagittal SPACE sequence configured with TR = 1500 ms, TE = 231 ms, flip angle $= 120^\circ$, matrix size = 320 × 304, in-plane resolution = 0.41 × 0.41 mm^2^, and slice thickness = 0.8 mm, similar to those used in related works  [21, 37–40]. At selected levels of the spinal canal, CSF flow-velocity data were acquired using a cardiac-gated, 2D phase-contrast MRI sequence perpendicular to the spinal canal axis. The sequence used through-plane velocity encoding to measure the velocity component normal to the imaging slice. Imaging parameters included TR = 23.78 ms, TE = 7.74 ms, flip angle $= 10^\circ$, matrix size = 256 × 205, in-plane resolution = 0.625 × 0.625 mm^2^, and slice thickness = 10 mm. Retrospective cardiac gating was achieved using peripheral pulse gating, with 40 cardiac phases reconstructed per cycle and an encoding velocity (VENC) of 8 cm/s. The measurements were conducted under free-breathing conditions. To assess the uncertainty associated with the PC-MRI velocity measurements, a complementary pulsatile-flow phantom study was performed using identical acquisition parameters. The methodology and results of this uncertainty quantification are provided in section S5 of the Supplementary Material.

**Fig. 1:**
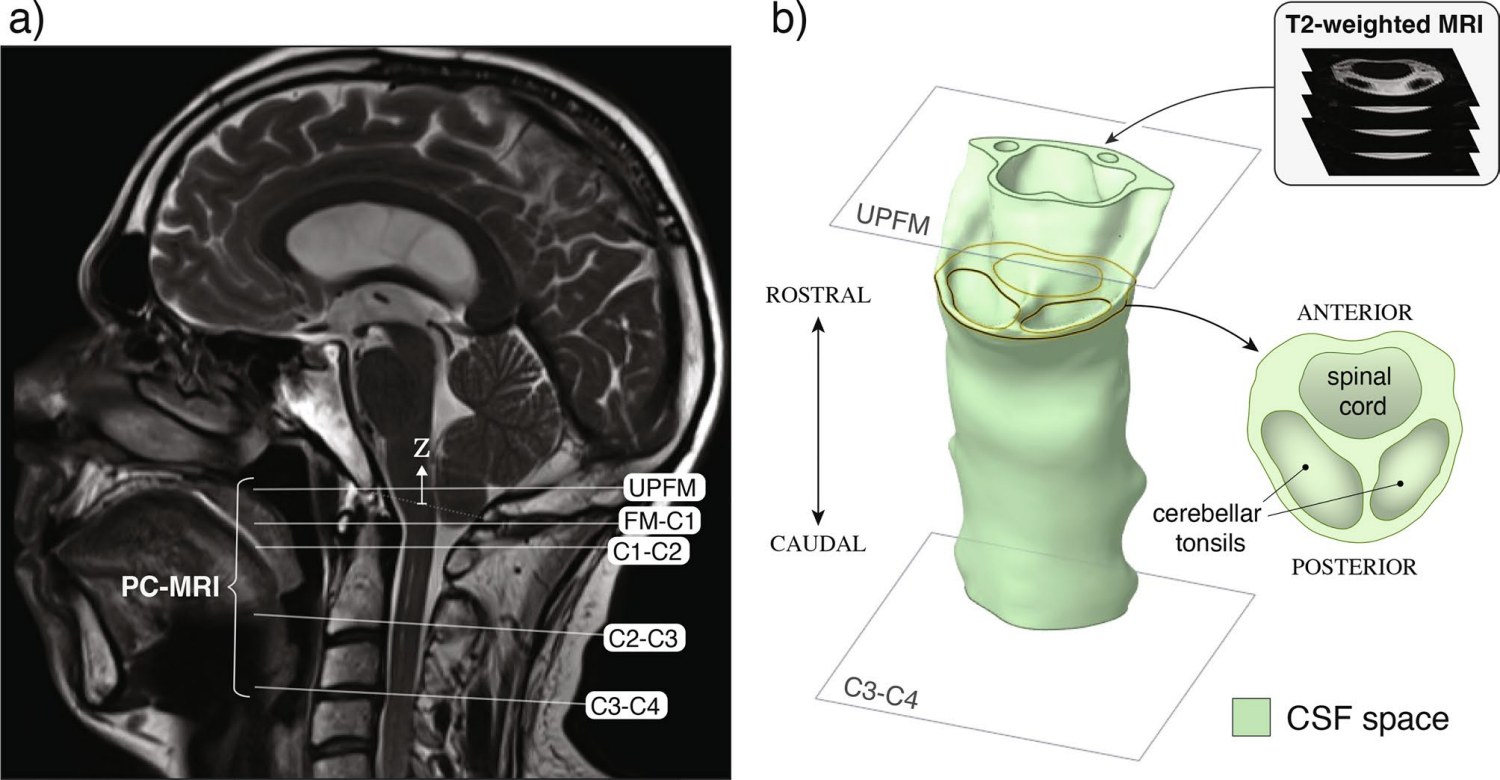
PC-MRI planes and CFD domain definition. (a) sagittal T2-weighted MRI of a patient with Chiari I malformation (CM-I), showing the locations of five PC-MRI measurement planes (UPFM, a plane above the foramen magnum; FM–C1, C1–C2, C2–C3, and C3–C4). (b) three-dimensional segmentation of the CSF space extracted from the T2-weighted MRI dataset, clipped by the UPFM and C3–C4 planes to define the computational domain for CFD simulations

Figure 1a shows the exact location and orientation of all PC-MRI measurements used in the analysis. As illustrated, five axial locations along the cervical canal were examined, referred to as UPFM, FM–C1, C1–C2, C2–C3, and C3–C4 according to adjacent anatomical landmarks. Here, UPFM denotes a location above the foramen magnum (FM), while FM–C1 corresponds to the region between the FM and C1. Additional sagittal MRI images illustrating relevant anatomical features, including the spinal syrinx and the descent of the cerebellar tonsils, are provided in Fig. S1 of the Supplementary Material.

### Image processing

#### T2-weighted: CSF space segmentation

T2-weighted images were used to segment the craniocervical CSF space, from the mid-cervical spine to 5–10 mm above the FM. Initial segmentation below the FM was performed automatically with the Spinal Cord Toolbox (SCT)  [41], while the region above was manually segmented in 3D Slicer  [42]. The complete segmentation was exported as a surface mesh for subsequent volume meshing. In addition, T2-weighted images, together with detailed anthropometric measurements, were used to generate the geometry of the nerve roots, as described below in Sect. 2.3.

#### PC-MRI: velocity field extraction

PC-MRI images were processed using an in-house MATLAB code  [43, 44]. For each measurement, the CSF region was manually delineated (ROI mask), and the velocity normal to the imaging slice was extracted over one cardiac cycle using phase and magnitude data.

Spatial filtering was applied to the velocity fields to reduce noise and remove artifacts. A 2D Gaussian filter ($\sigma =$ 0.5–1) was used to smooth high-frequency spatial variations, while outliers—defined as values deviating from the local 3 × 3 median by more than $\Delta =$ 20–40% of the VENC—were replaced with the corresponding median. Filter parameters (Δ and *σ*) were individually tuned for each measurement to preserve flow patterns while effectively removing localized artifacts. Finally, a pixel-wise time-periodic unwrapping procedure was applied to correct for phase aliasing.

Due to differences in acquisition timing and patient positioning, a registration step was required to align the PC-MRI data with the CSF segmentation from T2-weighted images. Each PC-MRI scan was first aligned using a rigid transformation, followed by a 2D symmetric diffeomorphic registration  [45] to deform the ROI mask to match the segmented CSF space. This ensured spatial correspondence for assigning boundary conditions and comparing CFD results with PC-MRI measurements.

### Anatomical detail: nerve roots and denticulate ligaments

**Fig. 2:**
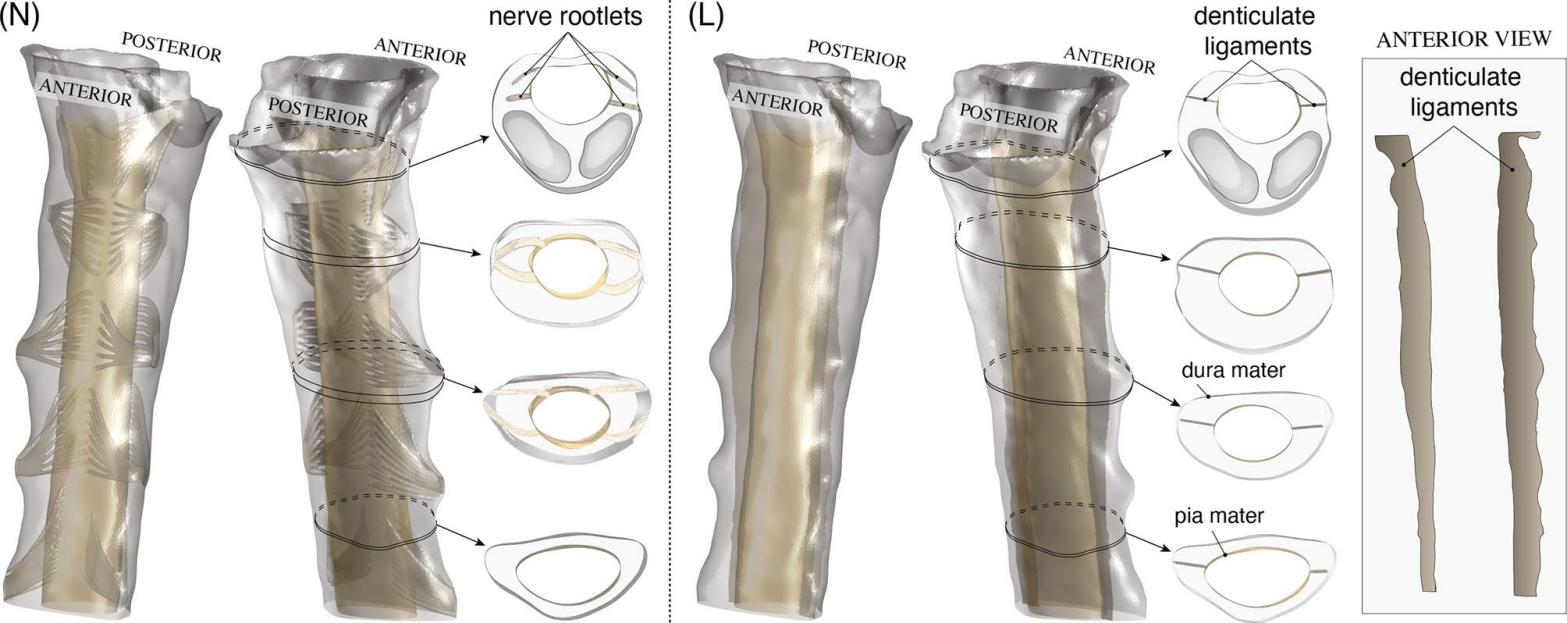
Microanatomical features incorporated into computational models. (N) Cervical nerve roots (C1–C4) Reconstructed from MRI and anatomical data, showing the rootlet arrangement at multiple axial levels. (L) Denticulate ligaments modeled as three-dimensional blade-like structures that run continuously along the pia mater and attach to the dura mater only at specific anchor points, partially separating the anterior and posterior compartments of the cervical canal, with geometry informed by ex vivo studies and PC-MRI velocity fields. An anterior view of the ligaments is also shown

Whereas the global CSF space–bounded by the spinal cord, dura mater, and cerebellar tonsils–is reconstructed directly from the patient’s T2-weighted MRI, the microanatomical features—nerve roots and denticulate ligaments—were modeled according to Parras-Martos et al. [46] by combining ex vivo anatomical data  [47–50] and MRI measurements. Given that our region of interest extends from the upper portion of the foramen magnum to vertebral levels C3–C4, only the cervical roots C1–C4 were included in the model. For each root we extracted the mean number of anterior and posterior rootlets, the intradural height of the anterior and posterior roots (rootlets that emerge from the spinal cord pair up into small subbundles, and these subbundles stack vertically to form the nerve root with a specific intradural height), and the rostral–caudal distance between the most rostral and the most caudal rootlets on the pia mater. Additionally, the diameter of rootlets was estimated from the root intradural height and the mean number of rootlets, knowing that the transverse area of the complete root should be the same as the transverse area of all the rootlets. Table 1 shows the anatomical measurements employed to build the geometric model of the cervical nerve roots. For every level (C1–C4) we list the mean number of posterior and anterior rootlets (*n*), the mean rootlet diameter (*d*_rlt_), the intradural height of the complete root (*h*_root_), and the rostral–caudal distance between the most rostral and the most caudal rootlets on the pia mater (*s*). Furthermore, the axial positions of the nerve roots (on the pia mater and the dura mater) relative to the foramen magnum, together with their azimuthal positions relative to the center of the spinal cord, were adjusted using MRI measurements. Finally, using all the data collected, we constructed a path line for each rootlet consistent with the observations of Mendez et al. [47]. The resulting geometry of the cervical canal with nerve roots is shown in Fig. 2(N).

**Table 1:** Anatomical measurements of the cervical nerve roots C1–C4 used in the present study (extracted from Mendez et al. [47]): *n* is the mean number of rootlets, *d*_rlt_ the mean rootlet diameter, *h*_root_ the intradural root height, and *s* the rostral–caudal length (distance between the most rostral and caudal rootlets) measured on the pia mater

Root	Posterior (dorsal)			Anterior (ventral)			Spacing *s* [mm]	
	n	*d*_rlt_ [mm]	*h*_root_ [mm]	n	*d*_rlt_ [mm]	*h*_root_ [mm]	posterior	anterior
C1	8	0.778	4.4	8	0.495	2.8	10.43	8.70
C2	8	0.778	4.4	8	0.495	2.8	10.43	8.70
C3	8	0.813	4.6	6	0.636	2.7	9.45	7.88
C4	9	0.800	4.8	8	0.520	2.9	12.00	10.50

The denticulate ligaments were represented as two thin three-dimensional laminae with a uniform thickness of 0.15 mm  [50], aligned with the spinal cord, continuously attached to the lateral surface of the pia mater and extending radially toward the dura mater. These laminae anchor to the dura mater at each vertebral level as well as at the foramen magnum, thereby yielding a total of four anchoring locations within the studied region of interest. Between consecutive vertebral anchor points, each ligament is linked only to the pia mater, projecting outward as a slender, blade-like strip. However, near the first anchor point, at the level of the foramen magnum, the blade has a large radial extent (see, e.g., Fig. 4 in Ceylan et al. [49]), which divides the anterior and posterior regions of the spinal canal, thereby enhancing the three-dimensional character of the velocity field, consistent with PC-MRI observations. Because the T2-weighted images do not have sufficient resolution to capture the exact location of the ligaments, their azimuthal position at a given vertical coordinate was adjusted with use of the velocity fields measured with PC-MRI—which, as will be shown, suggest the presence of a vertical barrier between the anterior and posterior compartments. Similarly, the radial extent of the ligament as a function of the axial coordinate was estimated in accordance with the descriptions of Tubbs et al. [48] and Ceylan et al. [49]. The reconstructed geometry including denticulate ligaments is shown in Fig. 2(L).

### Computational modeling

Using the segmented CSF space, the computational domain was defined in ANSYS SpaceClaim (2023 R1) by truncating the geometry at the UPFM and C3–C4 planes (Fig. 1b), and incorporating anatomical features such as nerve roots and ligaments when needed. Meshing was subsequently performed in ANSYS Fluent Meshing (2023 R1) using unstructured polyhedral elements. A local face sizing of 0.2 mm was applied to all solid boundaries—including the spinal cord, dura, tonsils, and, when applicable, nerve roots and ligaments—with five inflation layers added to better capture near-wall gradients. Proximity refinement was enforced between the spinal cord and surrounding structures to resolve narrow flow paths, ensuring a minimum of 10 cells across gaps. Final meshes ranged from 1 to 2 million elements, with higher counts required for anatomically detailed models. Mesh quality was verified to satisfy the minimum orthogonality threshold of 0.3 and the maximum skewness threshold of 0.7.

Flow simulations were performed using the finite-volume solver ANSYS Fluent (2023 R1), modeling CSF as a Newtonian fluid with density $\rho \simeq 1000\,\mathrm{kg/m}^3$ and kinematic viscosity $\nu \simeq 0.7 \times 10^{-6}\,\mathrm{m}^2/\mathrm{s}$. The associated Womersley number based on the characteristic canal width $h_c \simeq 4$ mm is ${\rm W}=(\omega h_c^2 /\nu)^{1/2} \simeq 12$, where $\omega =2 \pi/T$ is the angular frequency of the oscillations, with $T \simeq 1$ s representing the period of the cardiac cycle. For the typical maximum velocities $u_{\rm max} \simeq 6$ cm/s found in the cervical canal, the resulting peak value of the Reynolds number is ${\rm Re}=u_{\rm max} h_c/\nu \simeq 400$, with significantly smaller values characterizing the flow around individual anatomical features. As a result, the time-periodic motion remains stable, and a laminar-flow model is appropriate for describing the flow dynamics. The incompressible Navier–Stokes equations were solved with a time-implicit, second-order accurate scheme in both space and time, employing the COUPLED algorithm for velocity–pressure coupling. Each cardiac cycle, with period *T*, was discretized into 100 time steps, with 20 iterations per step. Simulations began with a steady-state initialization. The flow reached a time-periodic state after three cardiac cycles, consistent with the expected stability of the periodic solution, and the solution from the final cycle was used for analysis. Double-precision arithmetic was used, and convergence with respect to spatial and temporal resolution was verified through mesh refinement and time-step reduction. Sensitivity analyses on mesh size, time-step, and number of simulated cardiac cycles are summarized in Sect. 2.6 and presented in greater detail in Tables S1–S3 in the Supplementary Material.

Boundary conditions were informed by the patient-specific PC-MRI measurements shown in Fig. 1, which provided both temporally resolved velocity fields, at discrete pixel locations, and integrated flow rates. Specifically, at each time step, time-dependent boundary conditions were evaluated from PC-MRI-informed Fourier-reconstructed functions, given below in Eqs. (1) and (2). For models prescribing spatially resolved velocity profiles, the pixel-wise velocities (Eq. (1)), evaluated at the corresponding time, were imposed as Dirichlet conditions, with the solver interpolating them onto the boundary faces of the CFD mesh, whereas for models prescribing flow rate, the corresponding continuous flow-rate waveform (Eq. (2)) was used to compute the boundary velocity.

**Fig. 3:**
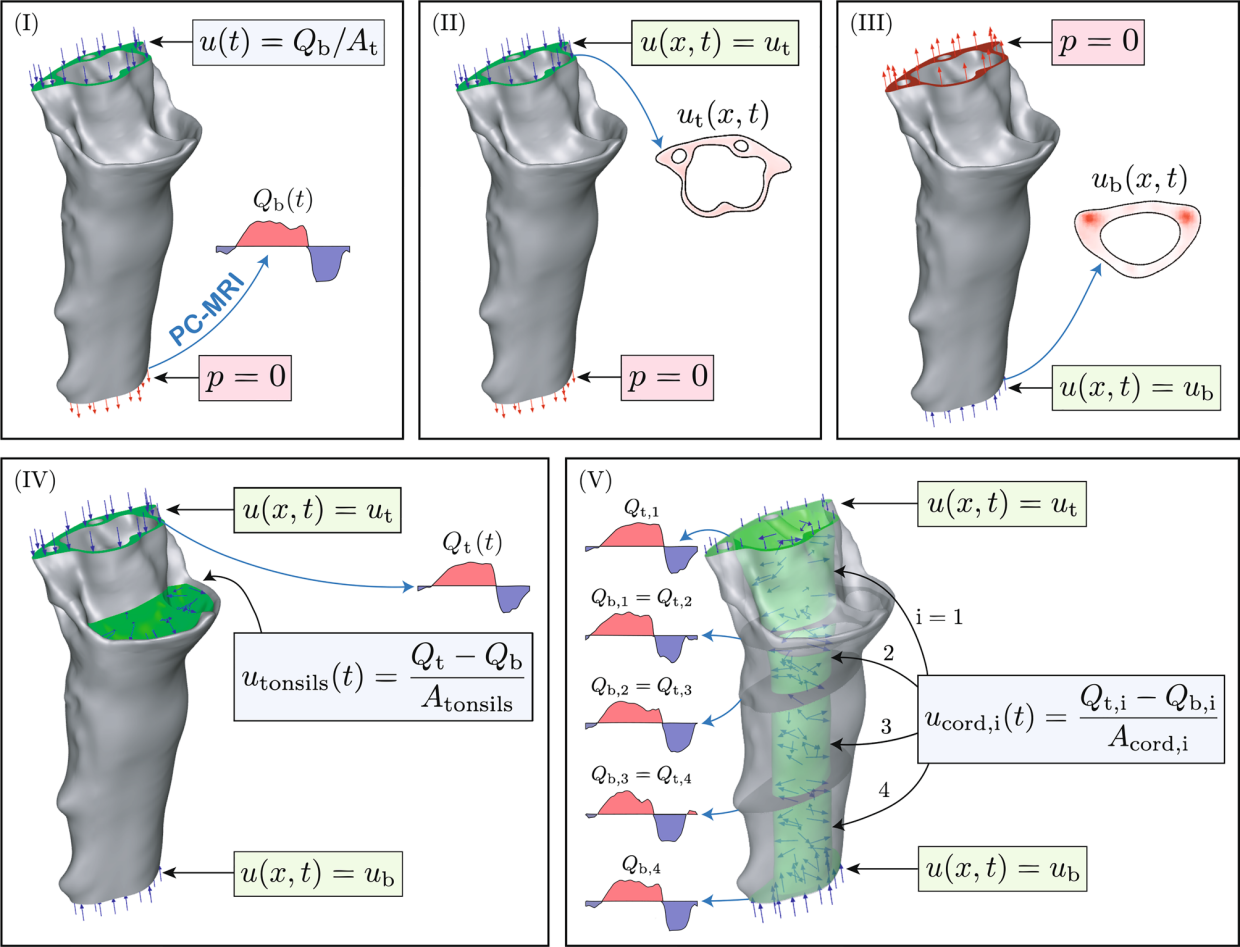
PC-MRI-based boundary condition configurations used in CFD simulations. Five boundary condition configurations were evaluated: (I) a spatially uniform, time-varying velocity imposed at the top boundary (UPFM), based on the flow rate measured at the bottom (C3–C4), with zero pressure (*p* = 0) prescribed at the latter boundary. (II) PC-MRI velocity profile imposed at the top, with *p* = 0 at the bottom. (III) PC-MRI velocity profile imposed at the bottom, with *p* = 0 at the top. (IV) PC-MRI velocity profiles imposed at both ends, with conservation of mass enforced via a prescribed penetration velocity normal to the tonsillar surface. (V) PC-MRI velocity profiles imposed at both ends, with additional inflow through the spinal cord surface adjusted to satisfy intermediate flow rate constraints. A no-slip condition ($\boldsymbol{u} = 0$) is applied at all solid walls where a penetration velocity is not explicitly prescribed. Subscripts *t* and *b* refer to the top (UPFM) and bottom (C3–C4) boundaries, respectively

**Fig. 4:**
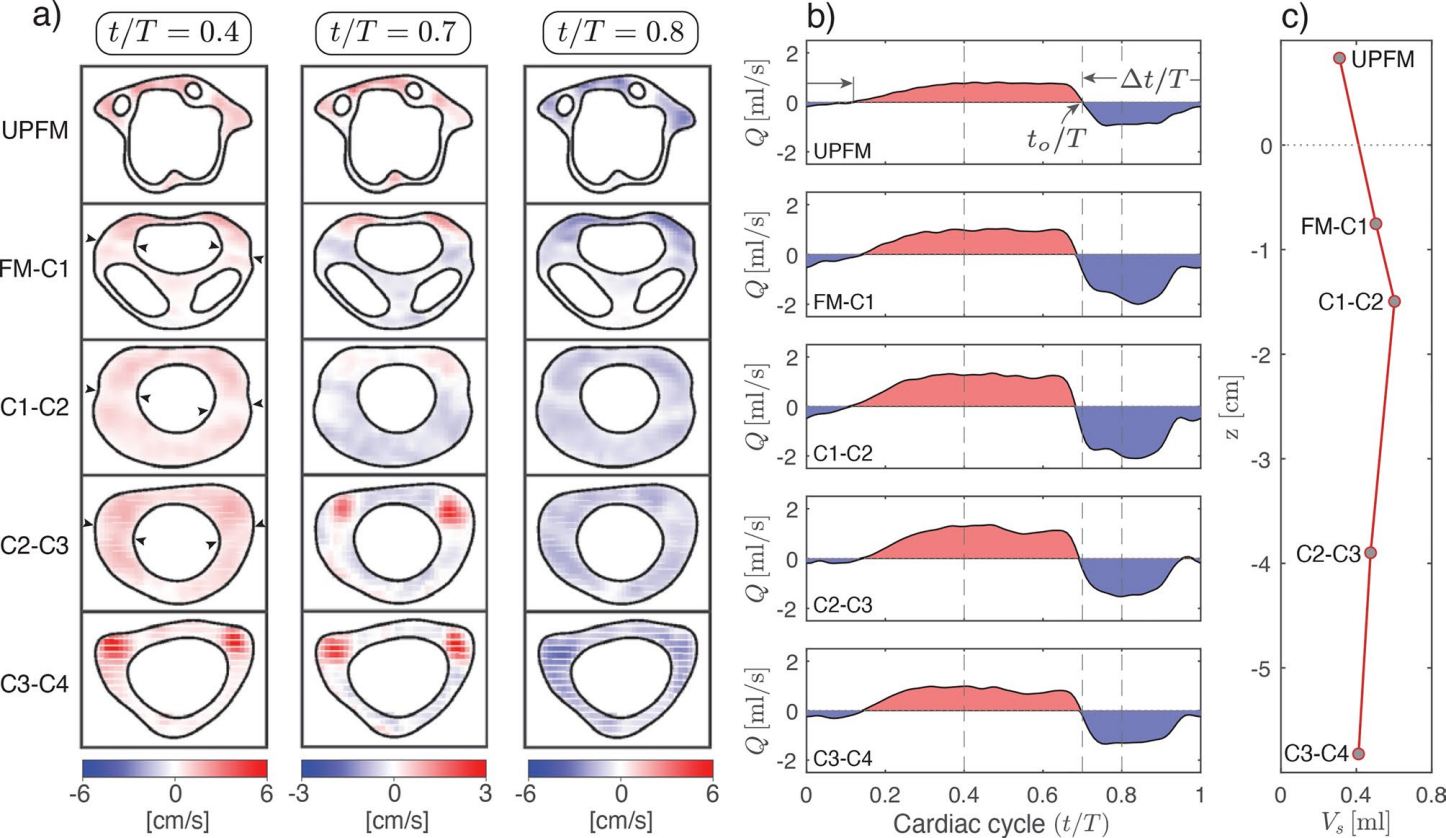
Cardiac-gated PC-MRI flow measurements of CSF flow. (**a**) Instantaneous velocity fields at three time points in the cardiac cycle, representative of peak rostral flow ($t/T = 0.4$), flow reversal ($t/T = 0.7$), and peak caudal flow ($t/T = 0.8$), where *T* is the cardiac period. Blue and red correspond to caudal and rostral flow directions, respectively. Arrows indicate regions where the velocity approaches zero near the midline. At flow reversal, a narrower color scale (−3 to 3 cm/s) is used to enhance the visibility of the low-magnitude velocities. (**b**) Evolution of the flow rate *Q* (Eq. (2)) over the cardiac cycle at each measurement plane, with vertical dashed lines added at the time points shown in panel (**a**). The normalized flow reversal time $t_o/T$ and duration $\Delta t/T$ of the caudal-flow phase are annotated in the uppermost location. (**c**) Stroke volume *V*_*s*_ (Eq. (3)) as a function of axial position *z*, with *z* = 0 at the foramen magnum

Five boundary-condition models, schematized in Fig. 3, were tested. Model (I) follows a conventional approach used in CM-I studies  [30–34], prescribing a time-varying but spatially uniform inlet velocity at the upper boundary (UPFM) and a zero-pressure condition at the outlet (C3–C4), using the flow-rate waveform measured at C3–C4 (last plot in Fig. 4b) to compute the time-varying boundary velocity. Models (II) and (III) impose spatially resolved PC-MRI velocity profiles at the upper or lower boundary, respectively, with zero pressure at the opposite end. Because zero-pressure boundary conditions can be sensitive to domain truncation, the robustness of models (I)–(III) with respect to axial domain length was explicitly evaluated by adding cranial and caudal flow extensions. The results of this analysis are presented in Sect. 2.6 and, in more detail, in Table S4 of the Supplementary Material.

Model (IV) removes the use of pressure boundary conditions altogether, prescribing PC-MRI informed velocity profiles at both boundaries and applying a compensatory normal velocity at the tonsillar surface to ensure mass conservation. The latter approach is motivated by recent experimental observations  [29] revealing that the net volume of fluid moved during a cardiac cycle at the foramen magnum (i.e., the stroke volume) is substantially lower than at the cervical canal and comparable in magnitude to the stroke volume of CSF displaced by tonsillar pulsations. Model (IV) is therefore intended as a simplified way to replicate the physiological effect of tonsillar motion on CSF dynamics, without explicitly solving the underlying fluid–structure interaction problem. Note that the use of a penetration velocity to replicate tissue deformation is not new–it has previously been adopted in numerical simulations of CSF flow in the spinal canal  [51]. Finally, model (V) allows us to satisfy the flow-rate constraints at all measured locations—top, bottom, and the three intermediate planes. Spatially resolved velocity profiles are prescribed at the top and bottom boundaries, whereas the flow-rate constraints at the intermediate planes (Fig. 4b) are enforced by introducing a normal velocity on the spinal cord surface. This additional velocity preserves mass conservation while adjusting the axial flow rate required to match the measurements. The normal velocity is distributed over four axial regions (between each pair of consecutive measurement planes) and is spatially uniform within each region, but varies in time so that the instantaneous flow rate at each intermediate location matches the corresponding PC-MRI waveform across the cardiac cycle, as illustrated in Fig. 3(V). This last model aims to replicate the effect of non-uniform tissue deformations in surrounding boundaries (spinal cord, dura, and tonsils), driven either by CSF pressure variations or by external mechanisms such as the pulsatile motion of blood vessels embedded within these tissues  [52, 53], which may induce local deformations coupled to the cardiac cycle. For models (IV) and (V), the pressure is not prescribed on the boundaries. Since pressure gradients drive the flow, the absolute static pressure is arbitrary. Therefore, a static pressure reference equal to zero is imposed at a single point inside the fluid domain, and the resulting velocity field is independent of the specific location chosen for this reference.

### Flow metrics

For each PC-MRI measurement slice, $u_{j,n}$ denotes the post-processed (filtered and de-aliased) velocity at pixel $j \in [1,\,2,\,\cdots,\, N_p]$ and time point $n \in [1,\,2,\,\cdots,\, 40]$, where *N*_*p*_ is the total number of pixels within the ROI mask, and the 40 time points correspond to the number of cardiac phases sampled over the cardiac cycle, of period *T*. For each pixel, the time-resolved velocity field is reconstructed by means of a Fourier approximation employing *M* = 20 modes, which is the maximum according to the Nyquist sampling theorem  [54, 55], expressed as

1 $$u_j(t) = \sum_{k=-M}^{M} c_{j,k} \, e^{\frac{2\pi {\rm i} k t} {T}},$$

where $c_{j,k}$ are the complex Fourier coefficients for pixel *j* and mode *k*. The reconstructed velocities are then used to compute the PC-MRI–inferred volumetric flow rate

2 $$Q(t) = \sum_{j=1}^{N_p} u_j(t)\, A_j,$$

with $A_j = {\rm d}x_j \times {\rm d}y_j$ denoting the area of each pixel. The stroke volume is subsequently calculated as

3 $$V_s = \frac{1}{2} \int_{t}^{t+T} |Q(t)|\,{\rm d}t.$$

Correspondingly, CFD simulations provide velocity fields at mesh nodes over $N_t = 100$ time steps per cardiac cycle. To enable direct comparison with PC-MRI data, these computed velocities are spatially averaged over pixel-sized bins of area ${\rm d}x_j \times {\rm d}y_j$, yielding binned velocities at each pixel location $j \in [1,\,2,\,\cdots,\, N_p]$. Temporally limited by the discrete time resolution of the simulation, these binned velocities are Fourier-approximated using *M* = 20 modes—consistent with the PC-MRI processing—resulting in the CFD-predicted, time-resolved velocity fields $\hat{u}_j(t)$.

To assess the agreement between CFD simulations and PC-MRI measurements, we compute the root-mean-square error (RMSE) of the velocity field at each measurement slice for selected time points. This slice-by-slice comparison allows for localized evaluation of model performance in both space and time. At a given time *t*, RMSE is computed from the reconstructed velocity fields $u_j(t)$ and $\hat{u}_j(t)$ as

4 $${\rm RMSE}(t) = \sqrt{\frac{1}{N_p} \sum_{j=1}^{N_p} \left[ u_j(t) - \hat{u}_j(t) \right]^2}.$$

To complement velocity comparisons, we introduce the impedance modulus *Z*_*L*_ and the longitudinal impedance (LI) as frequency-domain metrics that quantify resistance to oscillatory flow. Following Martin et al. [31], the impedance modulus for Fourier mode *k* is defined as

5 $$ Z_{L,k} = \left|\frac{\Delta p_k}{q_k}\right|,$$

where Δ*p*_*k*_ and *q*_*k*_ are the complex Fourier coefficients of the pressure drop $\Delta p(t)=\sum_{k=-M}^{M} \Delta p_k \, \exp(2\pi {\rm i} k \, t/T)$ and volumetric flow rate $Q(t)=\sum_{k=-M}^{M} q_k \, \exp(2\pi {\rm i} k \, t/T)$, respectively. While the flow-rate coefficients $q_k=\sum_{j=1}^{N_p}A_jc_{j,k}$ follow directly from Eqs. (1) and (2), $\Delta p(t)$ is computed as the difference between the area-weighted average pressure at the foramen magnum, $\overline{p}_{z=0}(t)$, and at a plane 2.5 cm caudal to the FM, $\overline{p}_{z=-2.5}(t)$, thereby yielding

6 $$\Delta p(t) = \overline{p}_{z=-2.5} - \overline{p}_{z=0}.$$

Consistent with prior analyses  [31], the longitudinal impedance LI is then computed by integrating the piecewise-linear curve obtained by connecting successive values of $Z_{L,k}$ over the frequency range $f = k/T \in [1,8]$ Hz. We note that the definition of $Z_{L,k}$ in Eq. (5) is applicable only when the flow rate is a function of time alone, i.e. independent of axial location. Accordingly, we will evaluate $Z_{L,k}$ and $\mathrm{LI}$ only for configurations (I)–(III), and omit cases (IV) and (V), for which $Z_{L,k}$ and LI are not uniquely defined, since the flow rate varies with axial location.

### Sensitivity analysis

To verify the numerical robustness of the simulations, additional calculations were performed to assess sensitivity to mesh resolution, time-step size, the number of simulated cardiac cycles, and the axial extension of the inlet and outlet when a zero-pressure boundary condition is applied.

Sensitivity with respect to mesh resolution, time-step size, and number of simulated cardiac cycles was assessed using the spinal canal geometry including nerve roots and denticulate ligaments under boundary-condition model (V). This configuration represents the most demanding case in terms of geometric complexity and boundary-condition enforcement. Three mesh resolutions (coarse/medium/fine), three temporal resolutions ($N_t = T/\Delta t = 50$, 100, and 200), and three cardiac-cycle counts (2, 3, and 10) were evaluated.

Sensitivity to domain truncation effects was assessed using boundary-condition model (I), which prescribes a spatially uniform velocity at the upper boundary and zero pressure at the lower boundary. Results obtained in the original computational domain were compared with those from an extended configuration in which 20 mm axial extensions were added at both the cranial and caudal ends (Fig. S2 in the Supplementary Material).

For all analyses, numerical sensitivity was quantified for both velocity and pressure. Velocity sensitivity was measured using a space–time *L*_2_ relative error (Eq. S1 in the Supplementary Material), while pressure sensitivity was assessed through the relative error in the peak pressure drop between the foramen magnum and an axial plane located 2.5 cm below (Eq. S2 in the Supplementary Material). Numerical results for all sensitivity analyses are provided in Tables S1–S4 in the Supplementary Material.

As a summary, these analyses demonstrate that the numerical results are robust with respect to all tested parameters. Mesh refinement and time-step size have the largest influence on the solution, with velocity and pressure errors decreasing systematically as spatial and temporal resolution are increased; for the selected medium mesh and temporal resolution of $N_t = 100$, velocity errors remain below 3.5% and pressure errors below 0.5%, providing a good compromise between accuracy and computational cost. The solution exhibits a weaker dependence on the number of simulated cardiac cycles, with a periodic steady state reached within three cycles, for which velocity errors fall below 0.35% and pressure errors below 0.05%. Similarly, the solution shows little sensitivity to the inclusion of axial domain extensions under zero-pressure boundary conditions, as such extensions produce velocity changes below 3% and relative variations in the peak pressure drop limited to 0.10%.

## Results

### Analysis of PC-MRI flow measurements

We begin by characterizing the craniocervical CSF flow of our CM-I patient using measurements acquired at five slices, including one above the foramen magnum (UPFM) and four within the cervical spine (FM–C1, C1–C2, C2–C3, and C3–C4). Cardiac-gated PC-MRI provided spatiotemporal distributions of the streamwise velocity–that is, the velocity component normal to each slice–from which we extracted the flow metrics presented in Fig. 4 and Table 2.

**Table 2:** Flow metrics derived from cardiac-gated PC-MRI measurements. For each measurement plane, the axial position *z* is given relative to the foramen magnum, along with the cardiac cycle period *T*. Reported parameters include the peak velocity $u_{\max}$ in the rostral $(+)$ and caudal $(-)$ directions. The table also lists the normalized onset time $t_o/T$ and duration $\Delta t/T$ of the caudal-flow phase, the maximum flow rates $Q_{\max}$, and the stroke volume *V*_*s*_ (Eq. (3))

Loc	*z* [cm]	*T* [s]	$u_{\max}$ [cm/s]		caudal-flow phase		$Q_{\max}$ [ml/s]		*V*_*s*_ [ml]
			(+)	(−)	$t_o/T$	$\Delta t /T$	(+)	(−)	
UPFM	0.83	1.14	2.67	3.77	0.72	0.39	0.80	0.93	0.31
FM–C1	−0.75	1.16	2.53	4.90	0.70	0.44	1.02	1.98	0.50
C1–C2	−1.50	1.14	1.76	3.42	0.70	0.40	1.33	2.08	0.60
C2–C3	−3.90	1.14	3.81	2.07	0.71	0.42	1.34	1.51	0.47
C3–C4	−5.82	1.13	6.13	4.04	0.71	0.43	0.97	1.33	0.41

Figure 4a shows visualizations of instantaneous velocity fields at three selected instants during the cardiac cycle, including peak rostral flow ($t/T \simeq 0.4$), flow reversal ($t/T \simeq 0.7$), and peak caudal flow ($t/T \simeq 0.8$). Note that at flow reversal, a different color scale (−3 to 3 cm/s) is applied to better resolve the low velocities characteristic of this phase. Let us begin by examining peak rostral and caudal flows, as the reversal phase presents a more complex behavior. At $t/T = 0.4$ and $t/T = 0.8$, flow in the upper regions (UPFM and FM–C1) is primarily restricted to the anterior subarachnoid space due to tonsillar obstruction. At C1–C2 and C2–C3, the flow becomes more evenly distributed across the canal, with near-zero velocities around the midline (indicated by arrows in the left column of Fig. 4a) likely attributable to the presence of nerve roots and/or denticulate ligaments. At the lowest level (C3–C4), bilateral anterior jets appear prominently during rostral flow, and although still present, they are less pronounced during caudal flow.

The flow reversal phase at $t/T = 0.7$ (second column of Fig. 4a) exhibits a particularly interesting bidirectional pattern. In the lower segments C3–C4 and C2–C3, anterolateral jets maintain rostral velocity while the rest of the canal has already transitioned to caudal flow. Immediately above, at levels C1–C2 and FM–C1, these rostral jets evolve into a more uniformly distributed rostral velocity in the anterior canal, while mid-to-posterior regions exhibit caudal flow. At the uppermost level (UPFM), the transition to caudal flow appears to lag, with flow still directed rostrally at the time point $t/T = 0.7$. This is consistent with the later onset of the caudal phase at this location, $t/T = 0.72$, as reported in Table 2. In relation to the bidirectional flow pattern observed at $t/T = 0.7$, it is worth noting that flow reversal is expected to occur first within the Stokes layers adjacent to the bounding surfaces. However, these near-wall features are not resolved in the PC-MRI images shown in the second column of Fig. 4a, because at the relatively large Womersley numbers (${\rm W} \sim 12$) characteristic of the cervical region, the Stokes layer thickness is comparable to the voxel size.

To complement the qualitative observations described above, Table 2 reports the peak rostral ($u_{\max}^{+}$) and caudal ($u_{\max}^{-}$) velocities at each axial location. Particular attention is given to the ratio between caudal to rostral velocities, which reveals a counterintuitive trend along the spinal axis. Just below the foramen magnum, at FM–C1 and C1–C2, peak caudal velocities are nearly twice as large as their rostral counterparts ($u_{\max}^{-}/u_{\max}^{+} \simeq 1.94$ at both locations), while a more balanced ratio of $u_{\max}^{-}/u_{\max}^{+} \simeq 1.41$ is found above the foramen magnum (UPFM). These values are comparable in magnitude to the caudal-to-rostral velocity ratio $u_{\max}^{-}/u_{\max}^{+} \simeq 1.76$ associated with the peak velocities $u_{\max}^{-}=6.0$ cm/s and $u_{\max}^{+}=3.4$ cm/s measured by Shah et al.  [39] at the foramen magnum in CM-I patients. At the lower cervical levels C2–C3 and C3–C4, however, the velocity ratio drops significantly, with caudal velocities approximately half the rostral values ($u_{\max}^{-}/u_{\max}^{+} \simeq 0.54$ and 0.66, respectively).

Across all slices analyzed in Table 2 and visualized in Fig. 4a, peak velocities are localized in the anterior region of the spinal canal and concentrate, at more caudal locations (particularly C3–C4), within the anterolateral jet-like structures described earlier. These jets are particularly dominant during rostral flow (left column of Fig. 4a), which explains why the maximum velocities reported in Table 2—exceeding 6 cm/s—occur during this phase, even though the corresponding peak rostral volumetric flow rate $Q_{\max}$ is lower than the caudal one, as will be discussed below. In contrast, during the caudal-flow phase (right column of Fig. 4a), the velocity field at the lower cervical levels is more uniformly distributed, leading to lower peak velocities. In fact, the highest velocity in the caudal direction, 4.9 cm/s, is observed just below the foramen magnum at FM–C1.

Integration of the PC-MRI velocity fields over the corresponding spinal canal cross-sectional area yielded the instantaneous volumetric flow rates shown in Fig. 4b. These curves reveal how both the magnitude and timing of CSF flow vary with axial location. Clearly, since the instantaneous flow rate would be identical everywhere should the canal be rigid, the changes reported here are indicative of lateral-boundary displacements producing time-dependent variations in local cross-sectional area. To quantify changes in magnitude, Table 2 includes peak rostral and caudal flow rates ($Q_{\max}^{+}$ and $Q_{\max}^{-}$). Peak instantaneous flow rates were observed at the mid-level measurement locations C1–C2 and C2–C3, with the values of 1.34 ml/s and 2.08 ml/s corresponding to peak rostral and caudal flows, respectively. These values decreased to 0.80 ml/s and 0.93 ml/s at the rostral extreme location (UPFM), and to 0.97 ml/s and 1.33 ml/s at the caudal end (C3–C4), corresponding to reductions by factors of approximately 1.68 and 2.24 at the upper end, and 1.38 and 1.56 at the lower end (for rostral and caudal flows, respectively). A similar pattern was observed in the stroke volume *V*_*s*_ (Fig. 4c), with the highest values at mid-levels and reductions toward the extreme locations. In particular, the maximum stroke volume of 0.6 ml at C1–C2 decreases by factors of 1.94 and 1.46 at the rostral and caudal ends, respectively.

Other flow metrics reported in Table 2 include the time of flow reversal *t*_*o*_ (i.e., the time at which the flow reverses from caudal to rostral) and the duration Δ*t* of the caudal-flow phase, both expressed as fractions of the cardiac period *T*. These values remain relatively consistent across all locations. The time of flow reversal $t_o/T$ ranges from 0.70 to 0.72, while the duration $\Delta t/T$ of the caudal-flow phase varies between 0.39 and 0.44. Likewise, the cardiac period *T* itself shows only minor variation across measurements, with values consistently near 1.14 s, indicating that the patient’s heartbeat remained very stable throughout the scan, which improves the reliability of each individual acquisition and ensures that all measurements were obtained under similar physiological conditions.

### Evaluation of modeling strategies

**Fig. 5:**
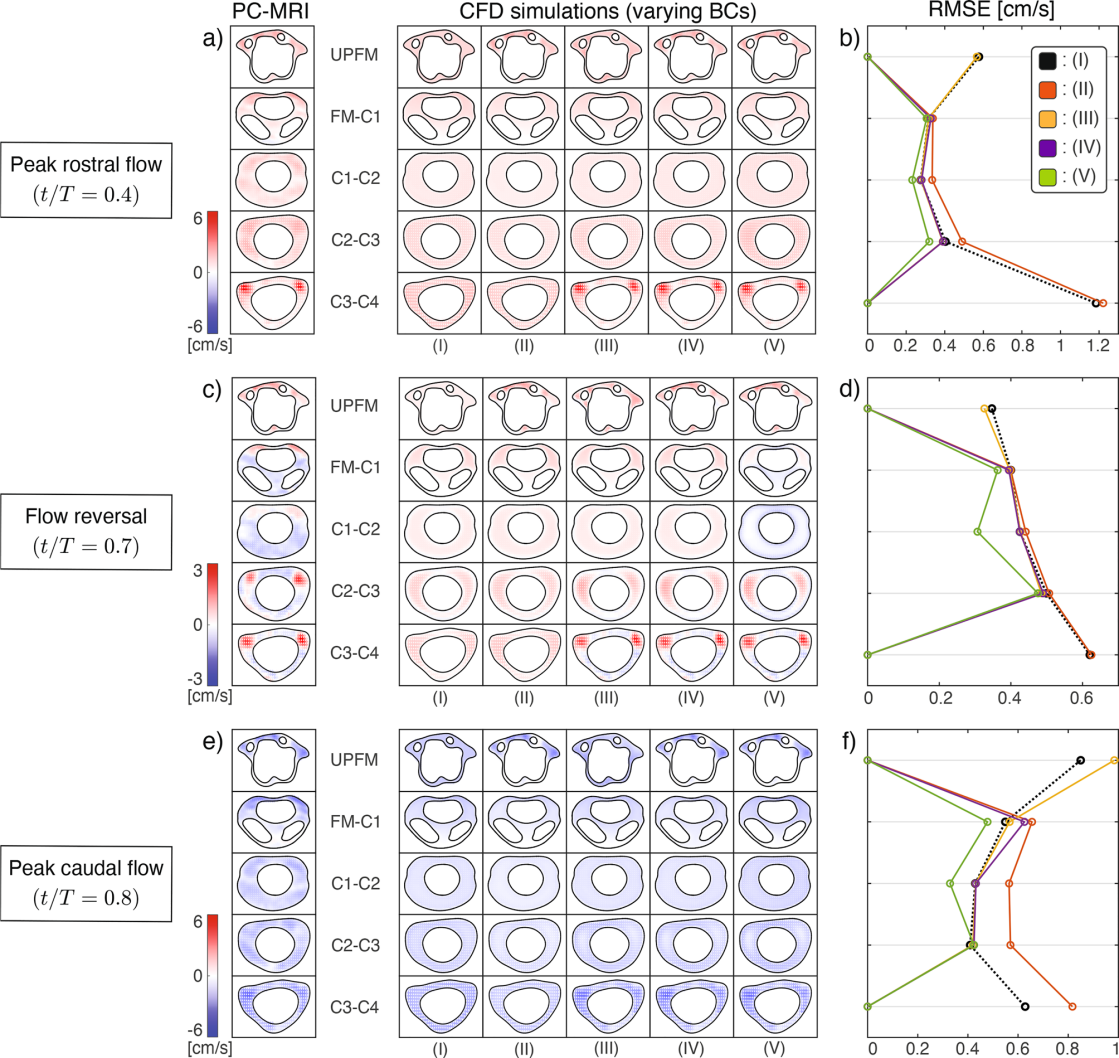
Impact of patient-specific boundary conditions on CFD agreement with PC-MRI data. CFD models (I)–(V) defined in Fig. 3 are evaluated against PC-MRI measurements at the five axial locations indicated in Fig. 1a. (a, c, e) show the velocity distributions at three characteristic points in the cardiac cycle: peak rostral flow ($t/T = 0.4$), flow reversal ($t/T = 0.7$), and peak caudal flow ($t/T = 0.8$). For each location and time, PC-MRI results are compared with CFD velocity fields (right columns), which have been spatially averaged over pixel-sized bins to match MRI resolution. At flow reversal, a reduced color scale (−3 to 3 cm/s) is used. For improved visualization at peak rostral and caudal flow, a reduced color-scale representation of $|u|$ is provided in Fig. S3. (b, d, f) display the root mean square error (RMSE), computed via Eq. (4), between each CFD model and PC-MRI data at the corresponding time points across all axial slices

To assess how boundary-condition selection affects the accuracy of the computational model, Fig. 5 compares the PC-MRI measurements with the CFD-predicted velocity fields obtained using the computational approaches (I)–(V) depicted in Fig. 3. The comparison spans all axial locations where PC-MRI measurements were acquired (UPFM to C3–C4; see Fig. 1a), at three time points in the cardiac cycle, representative of peak rostral flow ($t/T = 0.4$), flow reversal ($t/T = 0.7$), and peak caudal flow ($t/T = 0.8$).

In comparing the results of the different models, it is important to keep in mind the differences in flow rates that arise from the respective boundary conditions. For example, the flow rate at the UPFM section (rostral boundary) corresponds to that shown in the top panel of Fig. 4b for models (II), (IV), and (V), and to that shown in the bottom panel for models (I) and (III). Likewise, the flow rate at the C3–C4 section (caudal boundary) corresponds to that shown in the top panel of Fig. 4b for model (II), and to that shown in the bottom panel for models (I), (III), (IV), and (V). These distinctions influence the resulting RMSE errors, as discussed below.

As revealed by the spatial distributions of streamwise velocity in Figs. 5a, 5c, and 5e, differences between CFD models are particularly evident at the extreme planes (UPFM and C3–C4), where the boundary conditions are prescribed (see Fig. S3 in the Supplementary Material for an enhanced-contrast visualization at peak rostral and caudal flow). For models (II), (IV), and (V), the PC-MRI velocity profile is imposed at the upper boundary (UPFM), so agreement at that plane is expected by construction. Similarly, models (III), (IV), and (V) impose the measured velocity profile at the lower boundary (C3–C4), also resulting in exact agreement at that location. These matches simply reflect the use of PC-MRI data as input rather than a prediction of the model. In contrast, model (I) prescribes a spatially uniform velocity at UPFM, and models (II) and (III) use zero-pressure conditions at C3–C4 and UPFM, respectively, which leads to visible deviations from PC-MRI data at those boundaries.

At intermediate planes, the CFD-predicted velocity distributions tend to smooth out and become more uniform across the cross-section, regardless of the boundary conditions employed in the calculations. A notable exception is FM–C1, which consistently exhibits spatial variability due to tonsillar obstruction, resulting in diminished velocities in the posterior region. In addition, subtle differences in flow direction and magnitude—at a given time and location—reflect discrepancies in the instantaneous flow rate between models and PC-MRI measurements. These differences arise from the fact that PC-MRI flow rates vary between slices, as shown in Fig. 4b, whereas CFD models that rely on velocity boundary conditions prescribed at either one or both ends of the domain (I)–(IV), do not account for this spatial variability. For example, in model (II), the PC-MRI velocity profile is prescribed at UPFM and a zero-pressure condition is applied at C3–C4, resulting in a constant flow rate along the entire canal equal to that of the UPFM slice. A similar outcome occurs in models (I) and (III), where the flow rate is governed by the C3–C4 measurement. While model (IV) applies PC-MRI velocities at both ends and satisfies flow constraints at those locations, it still cannot match the measured flow rates at intermediate planes. By contrast, model (V) incorporates spatially distributed penetration velocity along the spinal cord, allowing the simulation to match the measured flow rate at every slice. This approach enables model (V) to better capture the spatial variability observed in PC-MRI data and achieve improved agreement across the domain.

To further evaluate model accuracy, Fig. 5b, d, and f report the RMSE error between CFD predictions and PC-MRI measurements, computed using Eq. (4) at each axial location and selected time point. As expected, the RMSE is identically zero at boundary locations where PC-MRI velocity is directly imposed. Among all models, configuration (V) consistently yields the lowest RMSE across slices and time points, indicating the best agreement with measured velocity fields.

It is instructive to compare these RMSE values with the intrinsic uncertainty of the PC–MRI measurements, which is quantified in section S5 of the Supplementary Material using a dedicated pulsatile phantom study performed under identical acquisition parameters. Considering the stroke volumes $V_s=[0.5,\,0.6,\,0.47]$ ml (Table 2) and the corresponding SAS cross-sectional areas $A_c=[2.32,\,2.23,\,1.54]$ cm^2^ at the intermediate measurement planes (FM–C1, C1–C2, and C2–C3), the time-averaged mean velocities are $v_c=[0.38,\,0.47,\,0.54]$ cm/s, obtained from $v_c = 2V_s/(T A_c)$ using a cardiac period of *T* = 1.14 s. For model (V), which provides the best agreement with PC–MRI, the corresponding time-averaged RMSE values are $\langle{\rm RMSE}\rangle=[0.39,\,0.34,\,0.32]$ cm/s. Even in this limiting scenario, the normalized errors ${\rm RMSE}/v_c=[1.03,\,0.72,\,0.59]$ are, in all cases, more than seven times larger than the normalized error of 0.081 obtained in the phantom uncertainty study (section S5 of the Supplementary Material). This comparison indicates that the discrepancies between CFD predictions and PC–MRI measurements cannot be attributed to measurement uncertainty alone, but instead reflect limitations in the modeling assumptions and boundary-condition formulations.

In addition, and consistent with the previous results, although increasing the level of complexity of the boundary conditions yields improved agreement with PC-MRI velocity data, important aspects of the flow remain elusive. For example, during peak caudal and rostral phases, the flow at the FM–C1 level is strongest in the anterior region, with a sharp anteromedial transition to weaker velocities. Regardless of the boundary conditions, this transition appears smooth in the CFD predictions, which fail to reproduce the observed sharp spatial gradient. Locations near the midline with near-zero velocities—caused by the presence of internal microanatomy and indicated with arrows in the left column of Fig. 4a—are also not captured, as expected for an unobstructed canal model. More notably, none of the models reproduce the bidirectional flow patterns observed in PC-MRI during flow reversal in the intermediate planes.

**Fig. 6:**
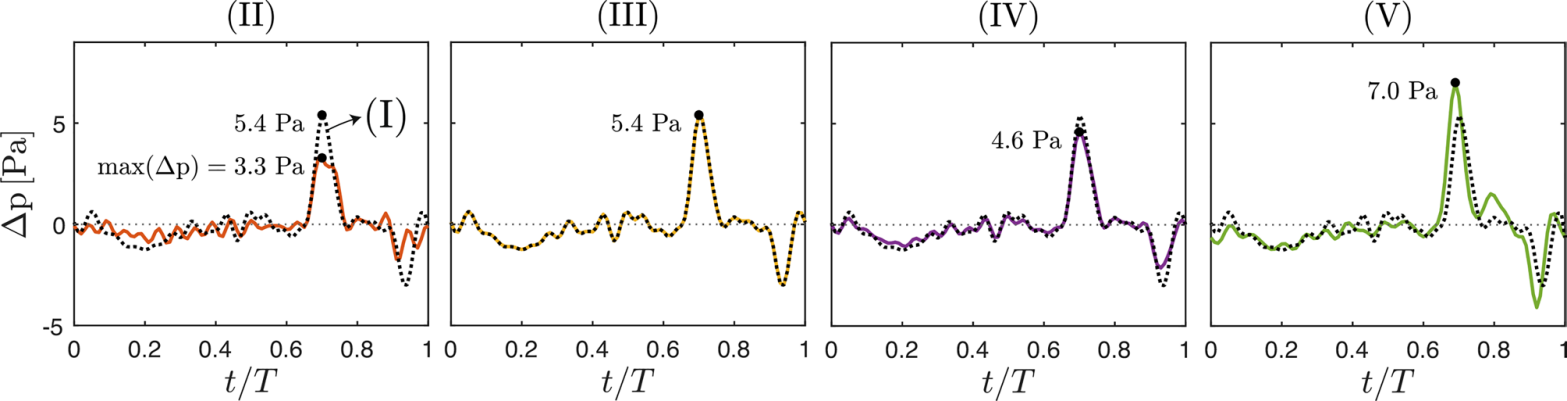
Evolution of the pressure drop Δ*p* (Eq. (6)) over the cardiac cycle, computed for boundary condition models (II)–(V) (solid lines) and compared with model (I) (black dotted line)

To evaluate the impact of boundary conditions on the pressure distribution, Fig. 6 shows the pressure jump Δ*p* between the foramen magnum and an axial plane 2.5 cm below, as defined in Eq. (6). This pressure drop has been used in previous studies to assess longitudinal impedance, which has been proposed as a hydrodynamic marker in CM-I  [31–33]. Among the five configurations, models (II) and (V) exhibit the most notable deviations. Model (II) produces a substantially lower peak pressure—3.3 Pa compared to 5.4 Pa in model (I)—representing a 39% decrease that is consistent with its reduced stroke volume ($V_s = 0.31$ ml vs. 0.41 ml in model (I)). In contrast, model (V), which provides the best agreement with PC-MRI data, yields a peak pressure drop of 7.0 Pa—30% higher than in model (I)—and introduces a slight phase lead relative to that baseline case.

These pressure values can be compared to those reported by Martin et al.  [31] in two adult male CM-I patients using a modeling approach similar to model (I). In their preoperative assessments, the peak pressure drops across the same region (from the foramen magnum to 2.5 cm below) reached 11.73 Pa (0.088 mmHg) for one patient (CP1) and 18.13 Pa (0.136 mmHg) for the other (CP2). These values are markedly higher—by factors of 2.2 and 3.4—than the peak pressure drop of 5.4 Pa obtained for our patient using model (I). Part of the explanation for the larger pressure drops reported in  [31] is the higher flow rates involved. This is evident when comparing the peak caudal and rostral flows of 1.33 and 0.97 ml/s in our patient (Table 2, C3–C4) with the values of 3.00 and 1.95 ml/s for CP1 (2.3 and 2.0 times higher), and 3.67 and 2.18 ml/s for CP2 (2.8 and 2.2 times higher). In addition to differences in flow rate, geometrical factors also contribute to the pressure losses, as reflected in the longitudinal impedance reported below.

The longitudinal impedance ${\rm LI}$ of models (I)–(III), calculated as described at the end of Sect. 2.6, was used to quantify resistance to oscillatory flow and assess its dependence on boundary conditions. The resulting values ${\rm LI} = 331$ (I), 336 (II), and 328 (III) dyn·s/cm^5^, differing by less than 3% from one another, are close to the value of 335 dyn·s/cm^5^ reported for CP2 in  [31] and substantially smaller than the 440 dyn·s/cm^5^ reported for CP1 in the same study, thereby revealing the latter case to have a higher degree of canal obstruction.

The small differences in LI values obtained from the three computational models stand in contrast to the variations in flow conditions. While model (II) prescribes the same flow-rate waveform (C3–C4) as model (I) but with a different inlet velocity distribution, model (III) imposes an alternative waveform (UPFM), which—as shown in Table 2—reduces the stroke volume by 24%. The results indicate that longitudinal impedance is largely insensitive to the details of the prescribed boundary conditions—whether the velocity profile is spatially uniform or resolved, and regardless of the flow-rate waveform, as previously pointed out by Shaffer et al. [32]. Furthermore, the near-invariance of ${\rm LI}$ across models with different flow-rate conditions suggests a predominantly linear relationship between pressure drop and flow rate. This points to a momentum balance governed mainly by viscous and unsteady inertial forces, while convective effects—expected to introduce nonlinearities—appear negligible within this frequency range.

In this connection, it is worth noting that the variation of the impedance modulus $Z_{L,k}$ with frequency is nearly linear for all three models (I)–(III), with the value of $Z_{L,k}/k$ differing in each case by about 5% over the entire range of frequencies considered in our analysis. This nearly linear proportionality reinforces the negligible role of convective terms and further indicates that pressure losses are balanced almost exactly by local acceleration, with frequency-independent viscous forces making only a comparatively minor contribution.

The observed dependences of $Z_{L,k}$ and LI are consistent with previous theoretical and numerical analyses of spinal-canal flow  [56–58], which have shown the flow dynamics to be fundamentally linear, governed by a balance between the local acceleration and the spatial pressure gradient, with a lesser contribution from viscous forces, as corresponds to motion at the large Womersley numbers ${\rm W} \simeq 12 \gg 1$ found in the cervical region. It is worth pointing out that, although in spinal-canal flow nonlinear effects stemming from convective acceleration are negligible in the first approximation, they produce a net streaming flow that dominates solute transport  [51, 59–61], as verified in recent direct numerical simulations  [46].

### Role of nerve roots and ligaments on flow patterns

**Fig. 7:**
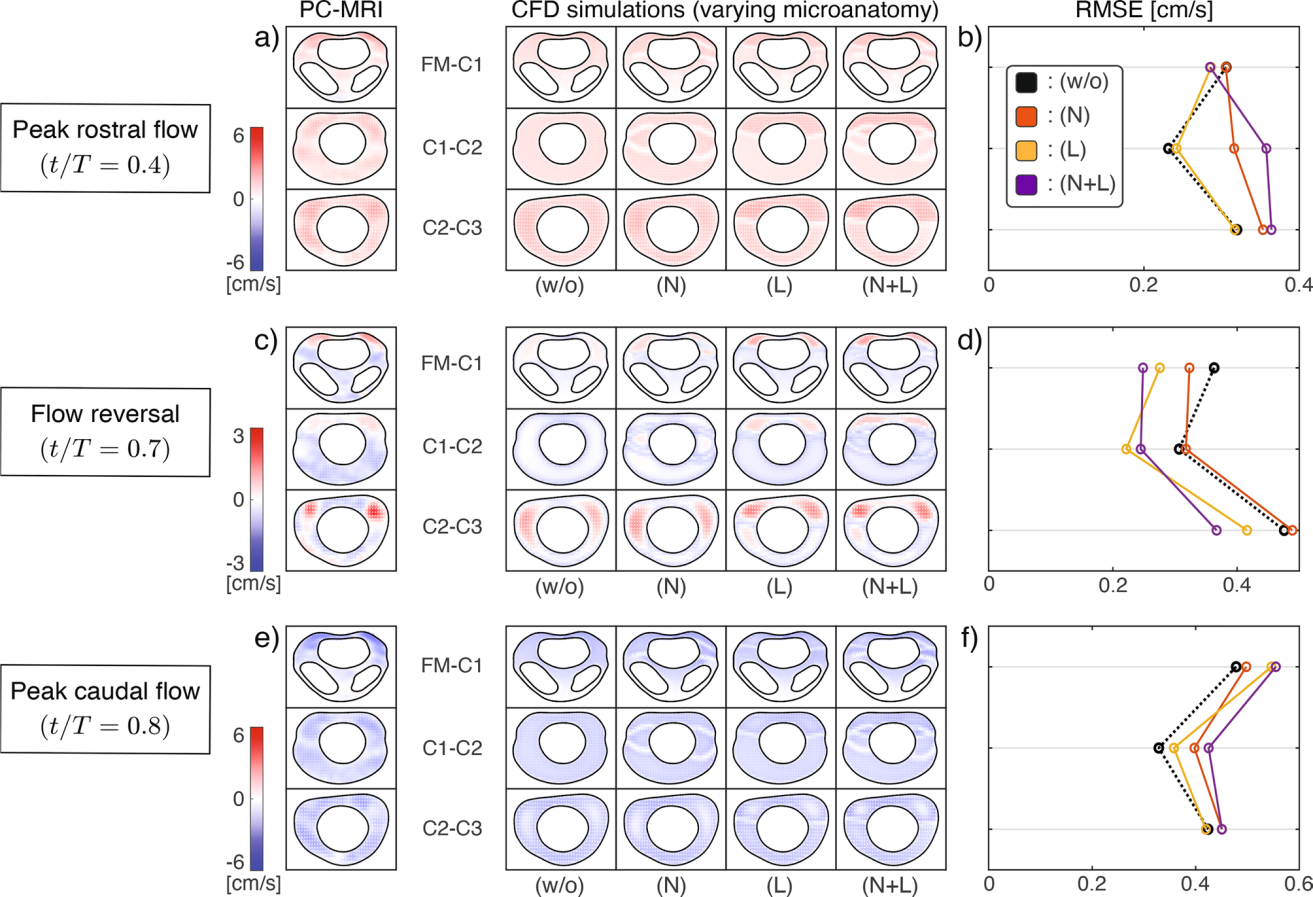
Impact of spinal microanatomy on CFD agreement with PC-MRI velocity data. CFD models incorporating nerve roots (N), ligaments (L), both (N+L), or neither (w/o) are evaluated against PC-MRI measurements at three axial locations (FM–C1, C1–C2, and C2–C3). All simulations use boundary condition model (V). (a, c, e) show velocity distributions at three key points in the cardiac cycle: peak rostral flow ($t/T = 0.4$), flow reversal ($t/T = 0.7$), and peak caudal flow ($t/T = 0.8$). For each location and time point, PC-MRI results are compared to CFD velocity fields (right columns), spatially averaged over 0.625 × 0.625 mm^2^ pixels to match MRI resolution. At flow reversal, a reduced color scale (−3 to 3 cm/s) is used. For improved visualization at peak rostral and caudal flow, a reduced color-scale representation of $|u|$ is provided in Fig. S4. (b, d, f) report the root mean square error (RMSE), computed using Eq. (4), between CFD predictions and PC-MRI data

**Fig. 8:**
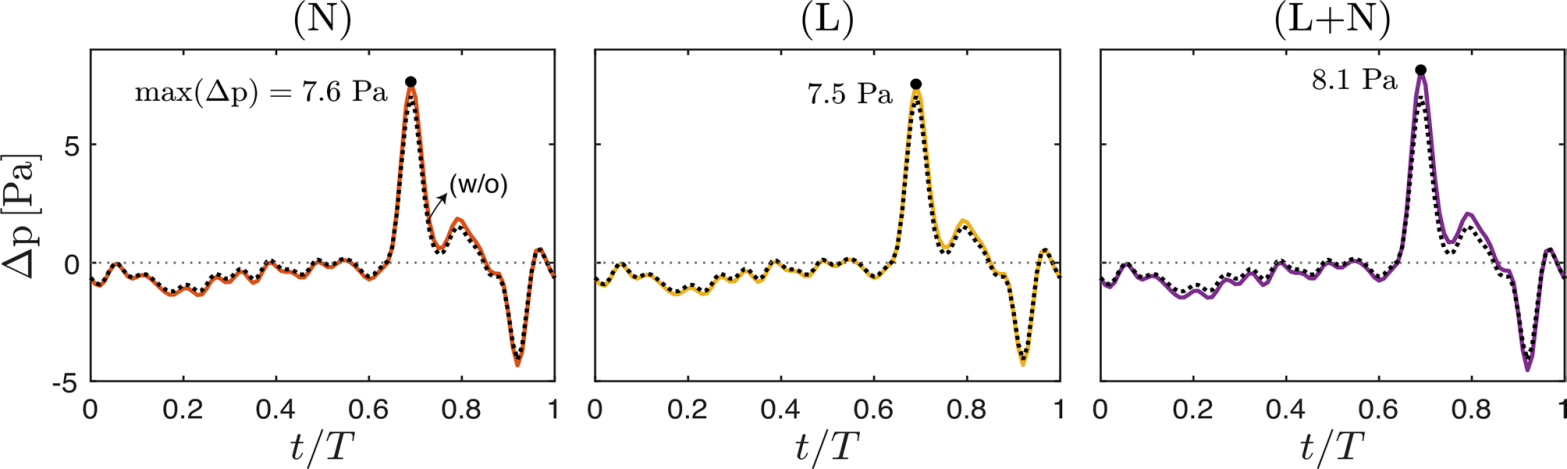
Evolution of the pressure drop Δ*p* (Eq. (6)) over the cardiac cycle, computed for the microanatomy configurations (N), (L), (N+L) (solid lines) and compared with the reference model without added structures (w/o, black dotted line). All simulations use boundary condition model (V)

Parallel to the analysis performed in Figs. 5 and 6 to assess the impact of boundary conditions in the unobstructed canal, Figs. 7 and 8 examine how the incorporation of microanatomical details affects velocity distributions and pressure drop in the cervical canal. Four configurations are tested, including one without microanatomical features (w/o) and three that incorporate nerve roots (N), ligaments (L), or both (N+L), with the corresponding anatomical structures shown in Fig. 2. Boundary conditions are prescribed using model (V) (see Fig. 3) in all cases, as it was shown earlier to yield the best agreement with MRI data. Since in model (V) the PC-MRI velocity is directly imposed at the two extreme slices UPFM and C3–C4, velocity results are shown in Fig. 7 only for the intermediate sections FM-C1, C1–C2, and C2–C3. Figure 8 presents the corresponding pressure drop Δ*p* between the foramen magnum and a plane 2.5 cm below for the different anatomical models. Figures S5 and S6, together with Table S5 in the Supplementary Material, present complementary results for the canal including nerve roots and denticulate ligaments (N+L) obtained using boundary-condition model (I).

As seen in Fig. 7a, c, and e, the addition of microanatomical features has a notable impact on the spatial distributions of streamwise velocity (see Fig. S4 in the Supplementary Material for an enhanced-contrast visualization at peak rostral and caudal flow). Locally, the no-slip boundary condition imposed on the surface of nerve roots and denticulate ligaments generates regions of near-zero velocity. On a broader scale, the ligaments act as physical barriers that restrict azimuthal flow and enhance anterior–posterior separation. This compartmentalization becomes most evident during flow reversal (Fig. 7c), when anterior flow is directed rostrally and posterior flow caudally, resulting in a bidirectional pattern that closely matches PC-MRI observations. The improved convergence at $t/T=0.7$ is reflected in the RMSE values in Fig. 7d, where configurations including ligaments—either with (N+L) or without (L) nerve roots—outperform the unobstructed case. For the peak-flow phases of Figs. 7a and  7e, however, inclusion of microanatomical structures does not improve velocity agreement with PC-MRI data according to the RMSE values reported in Fig. 7b and  7f, with the unobstructed case (w/o) yielding the lowest errors. Besides influences on velocity patterns, microanatomy has only a modest effect on the pressure drop Δ*p* (Fig. 8), with peak values increasing by 8.6% (N), 7.1% (L), and 15.7% (N+L) relative to the 7.0 Pa obtained for the unobstructed canal, while the waveform shape remains virtually unchanged. These findings are consistent with the 8.5–20% increase in pressure drop reported in previous numerical studies when including nerve roots and ligaments in the computations  [37, 46].

**Fig. 9:**
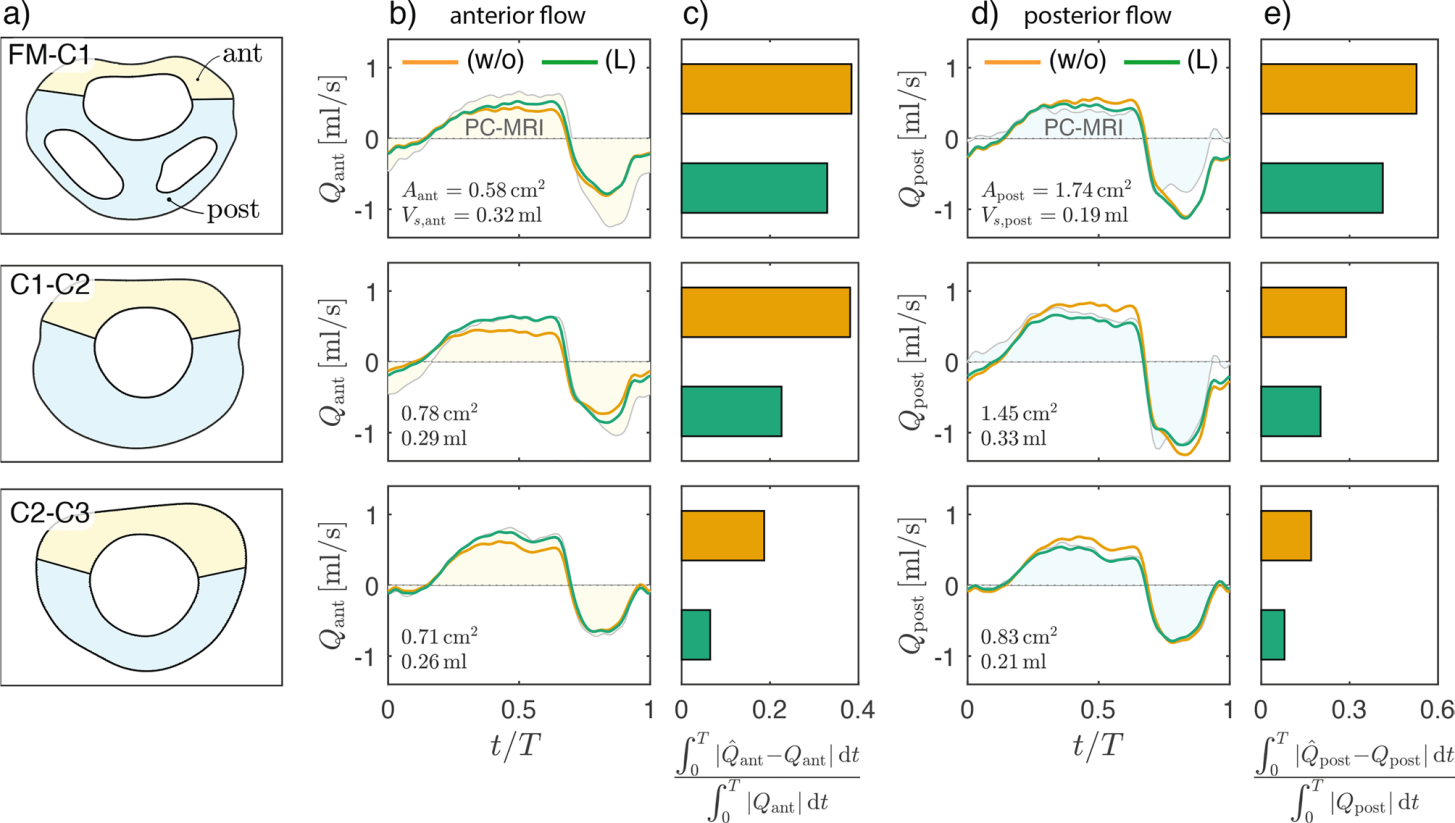
Anterior-posterior spinal canal flow rates at intermediate measurement planes. (**a**) Definition of anterior and posterior canal regions based on ligament location (see Fig. 2(L)). (**b, d**) Instantaneous flow rates over the cardiac cycle in the anterior (*Q*_ant_) and posterior (*Q*_post_) canal, respectively, predicted for the unobstructed canal (orange) and with ligaments (green), and compared with PC-MRI measurements (shaded areas). For each location, the anterior/posterior cross-sectional area *A* and the PC-MRI stroke volume *V*_*s*_ (Eq. (3)) are indicated. (**c, e**) Relative error between CFD-predicted $(\hat{Q})$ and PC-MRI-measured (*Q*) flow rates in the anterior and posterior canal, respectively. All computations use model (V) for boundary conditions

As previously noted, while adding nerve roots produces only local velocity changes, denticulate ligaments promote anterior–posterior flow separation within the spinal canal  [48]. A detailed assessment of regional flow helps quantify this effect. Specifically, Fig. 9b and 9d compare CFD-predicted flow rates in the anterior and posterior regions (as defined in Fig. 9a) for the unobstructed canal (w/o) and the canal with ligaments (L) against PC-MRI-measured values. Denoting CFD predictions by $\hat{Q}$ and PC-MRI-measured values by *Q*, the relative errors

7 $$\frac{\int_0^T \lvert \hat{Q}_{\mathrm{ant}} - Q_{\mathrm{ant}} \rvert\, {\rm d}t}{\int_0^T \lvert Q_{\mathrm{ant}} \rvert \,{\rm d}t}\quad\mathrm{and}\quad\frac{\int_0^T \lvert \hat{Q}_{\mathrm{post}} - Q_{\mathrm{post}} \rvert\, {\rm d}t}{\int_0^T \lvert Q_{\mathrm{post}} \rvert\, {\rm d}t},$$

given in Fig. 9c and 9e, show that including ligaments consistently improves agreement with PC-MRI measurements. For anterior flow, for example, the error decreases by factors of 1.17, 1.68, and 2.90 at FM–C1, C1–C2, and C2–C3, respectively, when ligaments are included. Further insight is provided by the regional cross-sectional areas and stroke volumes reported in Fig. 9b and 9d. Consistent with dominant anterior flow, even though the anterior area can be much smaller than the posterior one (e.g., $A_{\mathrm{ant}}=0.58~\mathrm{cm}^2$ vs. $A_{\mathrm{post}}=1.74~\mathrm{cm}^2$ at FM–C1) the corresponding stroke volume remains similar to or exceeds the posterior value (e.g., $V_{\mathrm{s,ant}}=0.32~\mathrm{ml}$ vs. $V_{\mathrm{s,post}}=0.19~\mathrm{ml}$). A separate observation is that the sum $V_{\mathrm{s,ant}} + V_{\mathrm{s,post}}$ can be slightly greater than the value *V*_*s*_ reported in Table 2 for the whole cross-section (e.g., $V_{\mathrm{s,ant}} + V_{\mathrm{s,post}}=0.62$ ml vs $V_s=0.60$ ml at C1–C2). This occurs because posterior and anterior flows have opposite directions during part of the cycle, so $\lvert Q_{\mathrm{ant}} + Q_{\mathrm{post}} \rvert < \lvert Q_{\mathrm{ant}} \rvert + \lvert Q_{\mathrm{post}} \rvert$, which makes $V_s < V_{\mathrm{s,ant}} + V_{\mathrm{s,post}}$ according to Eq. (3). Another notable feature is the different longitudinal variation of stroke volumes, with $V_{\mathrm{s,ant}}$ increasing steadily from 0.26 ml at C2–C3 to 0.32 ml at FM–C1, whereas $V_{\mathrm{s,post}}$ peaks at the intermediate location (0.33 ml at C1–C2), exceeding the values at both extremes (0.21 ml and 0.19 ml) by more than 50%.

**Fig. 10:**
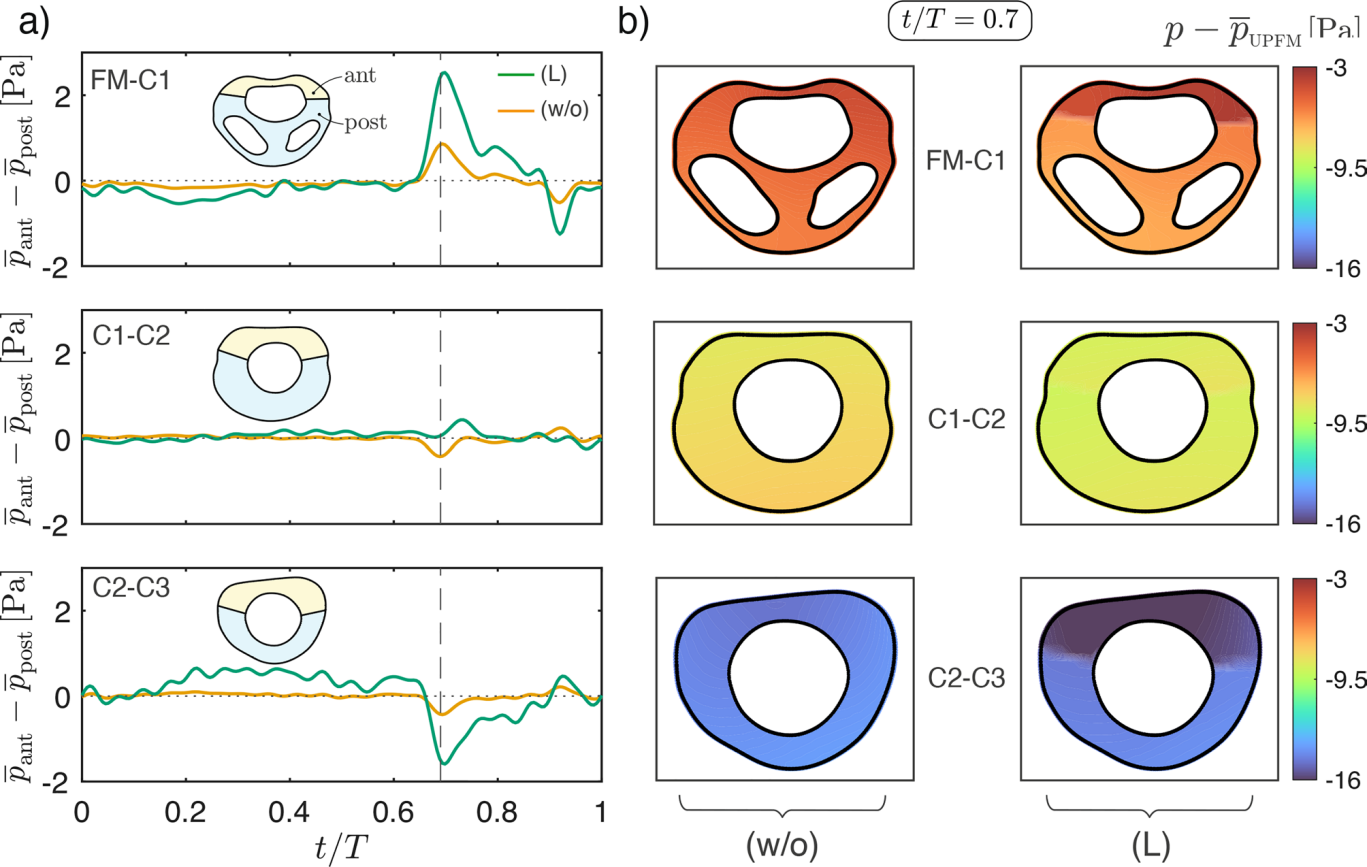
Anterior–posterior pressures at intermediate measurement planes. (**a**) Temporal evolution of the pressure difference between the anterior and posterior canal, $\overline{p}_{\rm ant} - \overline{p}_{\rm post}$, for the unobstructed canal (w/o) and with ligaments (L) at each intermediate location. (**b**) For time $t/T = 0.7$, spatial pressure distributions referenced to the mean pressure at UPFM ($\overline{p}_{\rm UPFM}$), with (w/o) on the left and (L) on the right. All computations use model (V) for boundary conditions

Flow compartmentalization has an impact on spatial pressure distributions, leading to larger spatial pressure nonuniformities within a given cross-section. As a metric to quantify this effect, one may use the difference $\overline{p}_{\rm ant} - \overline{p}_{\rm post}$ between the average values of the pressure in the anterior and posterior regions defined in Fig. 9a. The temporal evolution of this quantity over the cardiac cycle for cases with (L) and without (w/o) ligaments is shown in Fig. 10a for each intermediate measurement location. As expected, the presence of ligaments amplifies $\overline{p}_{\rm ant} - \overline{p}_{\rm post}$. For instance, at time $t/T=0.7$, corresponding to flow reversal, the addition of ligaments results in an increase from $\overline{p}_{\rm ant} - \overline{p}_{\rm post}=0.85$ Pa to 2.5 Pa at FM-C1. Correspondingly, at C2–C3 this pressure difference increases in magnitude from −0.4 Pa to −1.6 Pa when ligaments are added to the model. Notably, the sign change between these locations indicates that, at $t/T=0.7$, an anterior overpressure at FM–C1 transitions to an anterior underpressure at more caudal locations. This longitudinal change on the sign of $\overline{p}_{\rm ant} - \overline{p}_{\rm post}$ remains consistent throughout the cardiac cycle.

Figure 10b complements the previous observations by showing the spatial pressure distribution, referenced to the mean pressure at the UPFM, at time $t/T = 0.7$ of peak anterior–posterior pressure variations. Without ligaments, variations are smooth and remain below 1 Pa, whereas with ligaments, sharper anterior–posterior gradients emerge, with differences exceeding 2 Pa in some cases.

So far, we have analyzed the effect of microanatomy on flow using boundary-condition model (V). To assess the robustness of these findings with respect to the imposed boundary conditions, additional simulations were performed for the canal including microanatomical features using the simplest boundary-condition model, i.e. model (I). The corresponding results are reported in the Supplementary Material (Figs. S5–S6 and Table S5). Figure S5 presents velocity fields at the time of flow reversal and shows that, while agreement with PC-MRI is reduced compared to model (V), the inclusion of nerve roots and denticulate ligaments in model (I) yields improved agreement relative to unobstructed canal simulations computed using the same boundary-condition model. In particular, the velocity fields exhibit a more pronounced rostrally directed flow in the region anterior to the ligaments.

In addition, parallel to Fig. 9 for model (V), Fig. S6 examines anterior–posterior flow compartmentalization using model (I), revealing an increase in anterior flow in the presence of ligaments and improved agreement with PC-MRI-informed anterior flow rates, while posterior flow shows little or no improvement. These trends are further quantified in Table S5, where the inclusion of ligaments substantially increases the anterior-to-posterior stroke-volume ratio, bringing it closer to PC-MRI measurements. Together, these results indicate that dominant anterior flow emerges in the presence of denticulate ligaments even in the absence of spatially resolved inlet velocity information.

## Discussion

PC-MRI measurements of craniocervical CSF flow revealed characteristic features of CM-I  [9–11, 15], including anterior-dominant flow at the foramen magnum that evolved caudally into anterolateral jets as well as the emergence of bidirectional flow during rostral-to-caudal reversal. Our CFD analysis demonstrated that personalized boundary conditions alone cannot reproduce these flow patterns, and only with the inclusion of denticulate ligaments do the simulations capture the observed anterior–posterior flow separation and bidirectional reversal at the intermediate planes, with anterior flow directed rostrally and posterior flow directed caudally. Our mechanistic explanation is that posterior obstruction from tonsillar herniation in CM-I drives anterior-dominant flow, with denticulate ligaments acting as partial barriers that preserve this asymmetry along the cervical canal. In the absence of microanatomy, an increased azimuthal flow in the simulations homogenizes the downstream longitudinal velocity distribution, eliminating anterior–posterior separation, as observed in our unobstructed canal simulations.

Anterior–posterior spinal canal compartmentalization by denticulate ligaments has been described in several imaging-based studies  [48, 62, 63], but Pahlavian et al.  [37] remains the only work to link microanatomy to CM-I flow patterns, showing in CFD simulations that the presence of nerve roots and ligaments produced anterior-dominant CSF flow and jet-like structures. Their analysis, however, relied on simplified boundary conditions and lacked quantitative comparison with PC-MRI data—limitations that are addressed in the present study. Here we show, for the first time, that ligament-induced flow compartmentalization is accompanied by amplified anterior–posterior pressure differences. Such gradients may be clinically relevant to conditions like syringomyelia, whose pathogenesis is frequently associated with altered spinal canal pressure distributions  [30, 64–69]. However, establishing a direct clinical connection will require studies in larger cohorts—including CM-I patients with and without syringomyelia—and with more personalized reconstructions of denticulate ligament anatomy.

From a modeling perspective, while our results demonstrate that denticulate ligaments are essential for pathophysiologically realistic flow predictions in CM-I, nerve roots appear to play a minor role in oscillatory CSF dynamics. Within the limited set of axial planes analyzed, the inclusion of nerve roots primarily affected local velocity fields without altering the overall flow organization. They also produced a modest increase in longitudinal pressure drop, consistent with the behavior observed by Pahlavian et al.  [37]. In healthy subjects, by contrast, the more uniform flow at the level of the foramen magnum likely reduces the impact of ligament-induced compartmentalization, which may explain why nerve roots have historically received greater attention than ligaments in the literature  [37, 46, 70, 71].

While simulations using boundary-condition model (V), together with the inclusion of denticulate ligaments, allow us to reproduce in vivo flow patterns, the underlying modeling approach remains relatively complex and relies on multiple inputs that may not always be available. This motivates the need to identify which components are essential and which can be omitted in simplified models of CM-I hydrodynamics. Our results in Figs. 9–10 for model (V), complemented by those in Figs. S5–S6 and Table S5 in the Supplementary Material for model (I), show that although more personalized boundary conditions yield improved quantitative agreement with PC-MRI, spatially resolved boundary conditions are not fundamental for shaping anterior–posterior flow separation. Even when a spatially uniform velocity profile is prescribed, as in model (I), the presence of denticulate ligaments amplifies anterior flow and yields anterior-to-posterior stroke-volume ratios that remain close to those observed in vivo. At the same time, additional inputs—such as the supplementary inflow condition introduced in model (V) to approximate the effects of tissue deformation—do refine the quantitative agreement with PC-MRI and may be useful, when available, to account for the non-negligible contribution of tissue deformation to CSF flow. In this context, we acknowledge that our boundary-condition sensitivity analysis did not explore all relevant alternatives. In particular, Windkessel-type boundary conditions, as used in recent computational studies of CSF flow  [27, 72], could be applied at the inlet and outlet sections to incorporate the effects of cranial and spinal compliance on cervical flow dynamics. This promising approach warrants further investigation in future work.

The above discussion applies to moderate CM-I cases, such as the one examined here, in which obstruction and tissue displacement are relatively mild, as illustrated by the limited tonsillar descent of approximately 6.4 mm shown in Fig. S1b of the Supplementary Material. This highlights an important limitation of the present study. In more severe presentations—where obstruction approaches completeness and the effects of tonsillar pulsations become dominant—elastic effects are expected to play a much larger role, and the present modeling framework would need to be extended accordingly. Nevertheless, the current model may provide a foundation for more generalized approaches capable of addressing a broader spectrum of CM-I severity.

Other limitations of the present work should be noted. The model relies on a single patient and is restricted to the upper cervical region, precluding assessment of more caudal levels. Additional limitations arise from the use of PC-MRI data. The use of 2D PC-MRI provides only through-plane velocities, meaning that the imposed boundary conditions lack transverse components; incorporating in-plane velocity information further improves the fidelity of the inflow and outflow conditions. A related source of uncertainty arises from the presence of the vertebral arteries—visible as voids in the UPFM slice in Fig. 4—which, by carrying blood flow at much higher speeds than CSF, introduce local signal disturbances that add noise to the surrounding CSF and complicate image processing at this level. In addition, although these arteries are accounted for in the construction of the computational domain, transverse velocity components induced by arterial pulsations are not explicitly captured. Moreover, the limited spatial resolution affects both boundary condition specification and validation, with measured velocity fields needing to be smoothed to suppress artifacts, leaving discrepancies between CFD predictions and PC-MRI velocities obscured by measurement uncertainty. However, as quantified by the complementary phantom study presented in section S5 of the Supplementary Material, the uncertainty associated with PC–MRI velocity measurements is an order of magnitude smaller than the discrepancies observed between CFD predictions and in vivo data, indicating that these differences cannot be attributed to measurement uncertainty alone.

Another simplification that should be addressed in future work is the use of prescribed penetration velocities to replicate tissue displacement rather than fully solving the underlying fluid–structure interaction problem. This boundary motion, moreover, is likely associated with features of the measured flow that remain unexplained and may reflect additional physiological mechanisms. In particular, the counterintuitive peak stroke volume observed at intermediate levels (Fig. 4), which is consistent with findings from other studies  [29, 73] and exceeds the uncertainty expected from PC–MRI measurements as quantified by the complementary phantom study (section S5 of the Supplementary Material), is therefore unlikely to be a measurement artifact and suggests contributions from tissue displacement not solely driven by CSF pressure fluctuations. Although tonsillar motion could be a factor, sagittal CINE MRI (provided in the Supplementary Material) shows that pulsations are relatively small in this patient, suggesting that tonsillar motion alone is unlikely to fully account for the observed longitudinal changes in flow. Other mechanisms, such as pulsatile expansion of spinal or epidural vessels, may also contribute to the observed longitudinal changes in spinal canal flow.

A further limitation concerns the modeling of microanatomical features. This includes the modeling of nerve roots—which still rely on generic representations for unresolved finer aspects  [74]—and arachnoid trabeculae—which are omitted altogether as they are not visible in vivo with our current imaging capabilities—thereby limiting evaluation of their potential influence on CSF flow  [71, 75]. A similar limitation applies to denticulate ligaments, where reconstructions are constrained by the absence of in vivo imaging with sufficient resolution to capture their features on a patient-specific basis  [37]. Consequently, in this study denticulate ligaments were modeled from post-mortem data  [47–49], with PC-MRI–informed velocity fields used only to guide their location, leaving geometrical features such as the exact attachment points and radial extent uncertain. Achieving patient-specific characterizations of ligament geometry would not only enhance simulation fidelity but also enable direct analysis of potential links between ligament morphology and observed flow patterns, thereby improving mechanistic understanding of CSF dynamics in CM-I and syringomyelia. Until such data become available, progress can be made through sensitivity analyses of ligament geometry—including attachment site, radial extent, and orientation—together with realistic modeling of their elastic properties  [76, 77] rather than treating them as rigid boundaries. We leave these extensions for future work.

## Conclusions

The impact of common modeling assumptions on CFD predictions of CM-I hydrodynamics has been assessed using MRI data from a male patient with syringomyelia. To that end, the craniocervical CSF space was reconstructed from T2-weighted MRI, and 2D PC-MRI velocity fields were used both as boundary conditions and for model validation. Our comparative computational study examines the influence of different boundary conditions and varying levels of anatomical detail, thereby complementing prior investigations of cervical-canal flow  [37]. Our high-fidelity, patient-specific simulations show that including the denticulate ligaments yields CSF-flow predictions that more closely align with in vivo PC-MRI measurements, demonstrating their essential role as modulators of craniocervical CSF flow in CM-I. Their inclusion is recommended for physiologically realistic simulations in CM-I, and their potential clinical implications warrant further investigation.

## Electronic supplementary material

Below is the link to the electronic supplementary material.

Supplementary Material 1

Supplementary Material 2

## Data Availability

The MRI data for the patient included in this study cannot be shared publicly due to patient privacy restrictions. Additional data supporting the findings of this study, including processed datasets used to generate the figures, are available from the corresponding author upon reasonable request.

## Declarations

### Supplementary information

The Supplementary Material includes additional MRI images, numerical sensitivity analyses, and complementary CFD results assessing the effects of boundary conditions and microanatomy, as well as a dedicated phantom study to quantify the uncertainty of the PC–MRI measurements.
