# Supplementary material for: Patient-specific CFD modeling of CSF flow in Chiari I malformation: denticulate-ligament-induced compartmentalization explains flow patterns
Source: Fluids Barriers CNS. 2026 Mar 2;23:54. doi: 10.1186/s12987-026-00780-y (PMC13059373; doi:10.1186/s12987-026-00780-y)
Supplement: Supplementary file 2 — Supplementary Material 2 [file 12987_2026_780_MOESM2_ESM.pdf]

Supplementary Material

S1 Supplementary MRI images

This section provides additional sagittal MRI images of the Chiari I malformation (CM-I) patient analyzed in this study. The images shown in Fig. S1 illustrate the presence of a spinal syrinx (Fig. S1a) and the descent of the cerebellar tonsils below the level of the foramen magnum by approximately 6.4 mm (Fig. S1b). Note that two different sagittal planes are used: a mid-sagittal plane for panel (b) and a slightly off-midline (paramedian) plane for panel (a), as the syrinx is offset from the centerline and is not clearly visible in the mid-sagittal view.

S2 Sensitivity analysis

Additional simulations were performed to assess sensitivity to (i) mesh resolution, (ii) time-step size, (iii) the number of simulated cardiac cycles, and (iv) the axial extension of the inlet and outlet when a zero-pressure boundary condition is applied.

In all analyses (i–iv), numerical sensitivity was quantified for both velocity and pressure. The velocity error is defined through a space–time L_2 relative metric evaluated on the N_p pixels of the original PC-MRI image space, onto which the CFD velocity field is interpolated. The temporal dimension is discretized using $N_t = T/\Delta t$ uniformly spaced time points over one cardiac cycle of period T, where Δt is the time-step size. Given a tested solution (m) and a reference solution (ref), the dimensionless velocity error is

$$\varepsilon^{(u)} = \frac{\sqrt{\frac{1}{N_\mathrm{p}\, N_t} \sum_{i=1}^{N_\mathrm{p}} \sum_{t=1}^{N_t} \left[u^{(m)}(i,t) - u^{\mathrm{ref}}(i,t)\right]^2}}{\sqrt{\frac{1}{N_\mathrm{p}\, N_t} \sum_{i=1}^{N_\mathrm{p}} \sum_{t=1}^{N_t} \left[u^{\mathrm{ref}}(i,t)\right]^2}}. \tag{S1}$$

Sensitivity in pressure was evaluated by comparing the peak pressure drop $\Delta p_{\max}$ between the foramen magnum and an axial plane 2.5 cm below, yielding the corresponding relative error

$$\varepsilon^{(\Delta p_{\max})} = \frac{\left|\Delta p_{\max}^{(m)} - \Delta p_{\max}^{\mathrm{ref}}\right|}{\left|\Delta p_{\max}^{\mathrm{ref}}\right|}. \tag{S2}$$

To assess (i)–(iii), simulations were carried out on the spinal canal geometry including nerve roots and denticulate ligaments using boundary-condition model (V), which represents the most demanding configuration in terms of geometric and boundary-condition complexity. Three mesh resolutions (coarse/medium/fine), three temporal resolutions (N_t = 50, 100, and 200), and three cardiac-cycle counts (2, 3, and 10) were tested, with results summarized in Tables S1, S2, and S3.

To assess (iv), simulations were performed using boundary-condition model (I), which prescribes a spatially uniform velocity at the upper boundary and zero pressure at the lower boundary. Domain truncation effects were evaluated by comparing the original computational domain with an extended configuration in which 20 mm axial

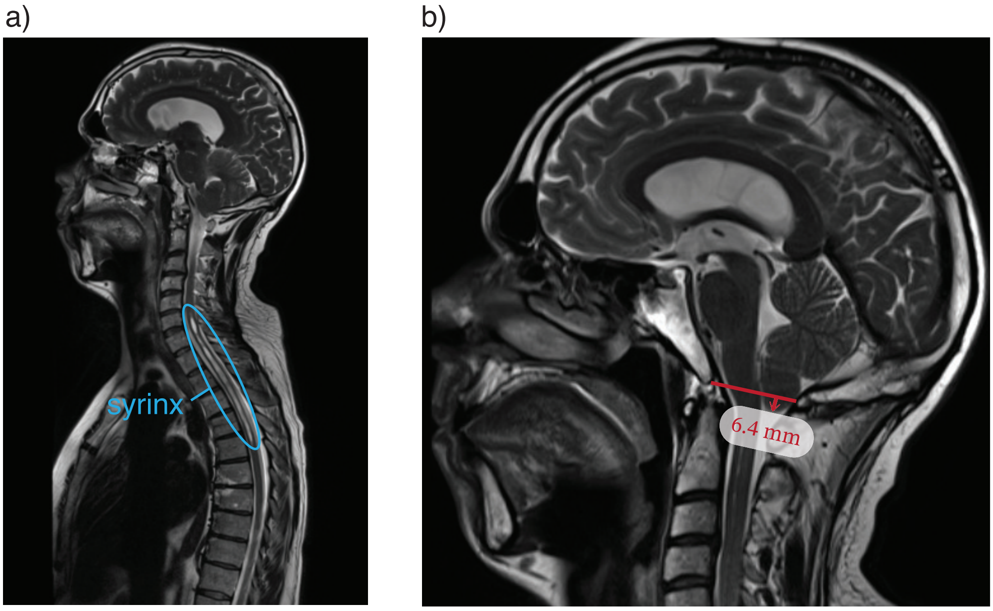

Fig. S1 Sagittal MRI images of the CM-I patient analyzed in this study. (a) Paramedian sagittal view of the cervicothoracic spine highlighting the presence of a syrinx within the spinal cord. (b) Mid-sagittal cranial view illustrating the descent of the cerebellar tonsils below the level of the foramen magnum, with a measured tonsillar herniation of 6.4 mm.

extensions were added in both the cranial and caudal directions (Fig. S2), with results reported in Table S4.

S2.1 Mesh sensitivity analysis

Table S1 Mesh-sensitivity analysis. The simulation with $\Delta x = 0.15$ mm is used as the reference for all errors.

Δx (mm)	Cells [$\times 10^6$]	$\varepsilon^{(u)}$ (%)			$\varepsilon^{(\Delta p_{\max})}$ (%)
		FM	C1–C2	C2–C3	
0.40	0.45	5.79	5.04	4.95	0.88
0.20	1.85	1.49	1.18	0.98	0.02
0.15	3.46	—	—	—	—

A mesh-sensitivity analysis was conducted using three spatial resolutions, with grid spacings $\Delta x = 0.40$, 0.20, and 0.15 mm (corresponding to the minimum element size), resulting in approximately 0.45, 1.85, and 3.46 million cells, respectively. The finest mesh ($\Delta x = 0.15$ mm) was taken as the reference solution for computing the velocity and pressure errors defined in Eqs. (S1) and (S2), with results reported in Table S1. Velocity errors decrease from about 5–6% on the coarse mesh to below 1.5% on the

medium mesh across all locations. Similarly, the relative error in the peak pressure drop $\Delta p_{\max}$ is below 1% for the coarse mesh and becomes negligible (0.02%) for the medium mesh. These results indicate that the solution is well converged with respect to spatial resolution, with the medium mesh providing a good compromise between accuracy and computational cost.

S2.2 Time-step sensitivity analysis

A time-step sensitivity analysis was performed using three temporal resolutions, corresponding to $N_t = 50$, 100, and 200, with the finest temporal resolution ($N_t = 200$) taken as the reference solution. Velocity and pressure errors were computed using the metrics defined in Eqs. (S1) and (S2), with results summarized in Table S2. For the velocity error evaluation, solutions were compared at instants of time corresponding to the coarsest temporal resolution ($N_t = 50$), thereby avoiding temporal interpolation. Velocity errors decrease systematically with temporal refinement, from 6–10% for $N_t = 50$ to below 3.5% for $N_t = 100$ across all axial locations. Similarly, the relative error in the peak pressure drop $\Delta p_{\max}$ is reduced from about 11% for $N_t = 50$ to 0.33% for $N_t = 100$. These results indicate that the solution is well converged with respect to the time-step size, with $N_t = 100$ providing a good compromise between accuracy and computational cost.

Table S2 Time-step sensitivity analysis. The simulation with $T/\Delta t = 200$ is used as the reference for all errors.

$N_t = T/\Delta t$	$\varepsilon^{(u)}$ (%)			$\varepsilon^{(\Delta p_{\max})}$ (%)
	FM	C1–C2	C2–C3	
50	8.09	10.17	6.15	10.57
100	2.76	3.46	2.10	0.33
200	—	—	—	—

S2.3 Cycle-sensitivity analysis

A cycle-sensitivity analysis was conducted to assess the influence of the number of simulated cardiac cycles on the computed velocity and pressure fields. Simulations including 2, 3, and 10 cardiac cycles were performed, with the solution obtained after 10 cycles taken as the reference. Velocity and pressure errors were evaluated using the metrics defined in Eqs. (S1) and (S2), with results reported in Table S3 revealing that the solution exhibits only a weak dependence on the number of simulated cycles. Velocity errors decrease from about 1% for two cycles to below 0.35% for three cycles across all axial locations. Similarly, the relative error in the peak pressure drop $\Delta p_{\max}$ remains below 0.1% for two cycles and decreases to 0.02% for three cycles. These results indicate that a periodic steady state is rapidly attained and that simulating three cardiac cycles is sufficient to achieve converged velocity and pressure fields.

Table S3 Cycle-sensitivity analysis. The simulation with 10 cycles is used as the reference for all errors.

Cycles	$\varepsilon^{(u)}$ (%)			$\varepsilon^{(\Delta p_{\max})}$ (%)
	FM	C1–C2	C2–C3	
2	1.31	0.76	1.01	0.06
3	0.27	0.33	0.18	0.02
10	—	—	—	—

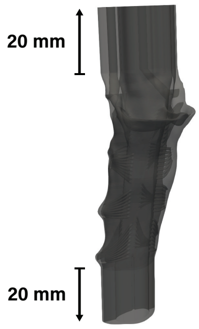

Fig. S2 Geometry of the craniocervical CSF space, including denticulate ligaments and nerve roots, with 20 mm extensions added cranially and caudally.

S2.4 Domain extensions

A domain-extension sensitivity analysis was performed to assess the influence of axial domain extensions when applying a zero-pressure boundary condition. Simulations were conducted using the original geometry and an extended domain in which 20 mm were added both cranially and caudally (Fig. S2); both configurations include nerve roots and denticulate ligaments, and the extended-domain solution was taken as the reference. Velocity and pressure errors were evaluated using the metrics defined in Eqs. (S1) and (S2), with results reported in Table S4. Velocity errors remain below 3% across all axial locations in the absence of domain extensions, while the relative error in the peak pressure drop $\Delta p_{\max}$ is limited to 0.10%. These results indicate that the solution exhibits only weak changes when axial domain extensions are included and that their effect can be neglected without significantly affecting the results.

Table S4 Domain-extension sensitivity. The simulation with 20 mm extensions (Fig. S2) is used as the reference for all errors.

Extension	$\varepsilon^{(u)}$ (%)			$\varepsilon^{(\Delta p_{\max})}$ (%)
	FM	C1–C2	C2–C3	
No extension	2.81	1.81	2.27	0.10
20 mm extensions	—	—	—	—

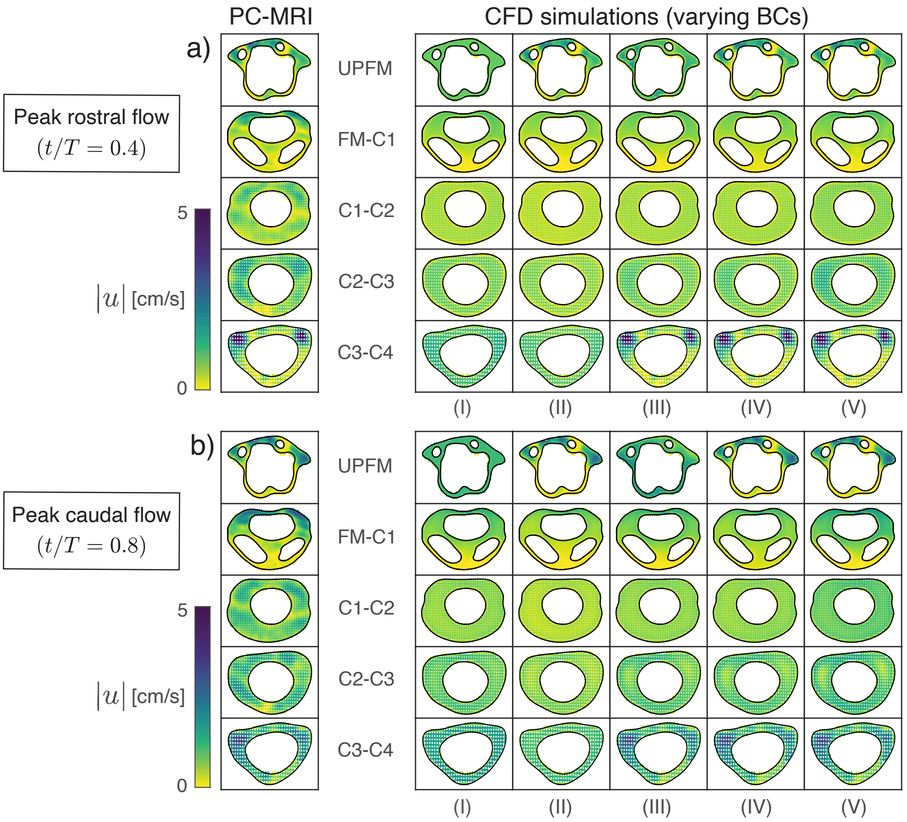

Fig. S3 Supplement to Fig. 5, assessing the impact of boundary conditions, showing $|u|$ with enhanced contrast. Same layout as Fig. 5, but using a reduced color scale (0–5 cm/s). Results are shown at (a) peak rostral ($t/T = 0.4$) and (b) peak caudal ($t/T = 0.8$) flow for PC-MRI (left) and CFD models (right).

S3 Supplementary figures for flow visualization

To allow for a clearer visualization of differences between boundary-condition models and microanatomical configurations, we provide additional figures in which the velocity field is represented using an alternative color scaling. In particular, the absolute velocity magnitude is shown using a reduced range (0–5 cm/s, instead of the symmetric range -6 to 6 cm/s), which enhances contrast in regions of low-to-moderate velocities that are less apparent when using a symmetric color scale. Figure S3 supplements Fig. 5 of the main manuscript, while Fig. S4 supplements Fig. 7. These figures are intended to aid qualitative comparison between CFD predictions and PC-MRI measurements.

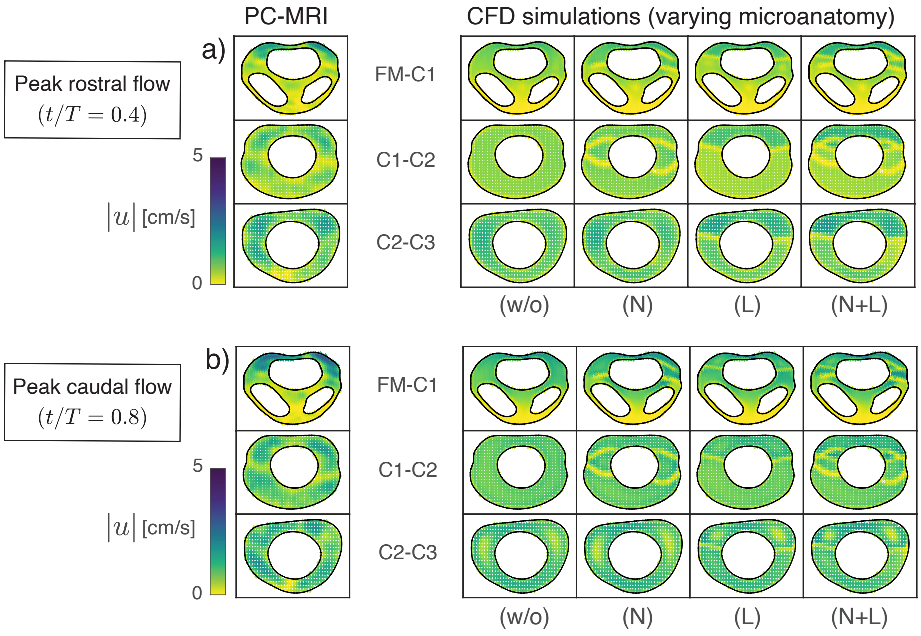

Fig. S4 Supplement to Fig. 7, assessing the impact of microanatomical features, showing $|u|$ with enhanced contrast. Same layout as Fig. 7, but using a reduced color scale (0–5 cm/s). Results are shown at (a) peak rostral ($t/T = 0.4$) and (b) peak caudal ($t/T = 0.8$) flow for PC-MRI (left) and CFD models (right).

S4 Evaluating canal with microanatomy using boundary condition model (I)

In the main manuscript, boundary-condition model (V) was used to asses the effect of adding microanatomical features, as it incorporates the highest level of patient-specific information available. Here, we complement those results by considering boundary-condition model (I), and assessing the effect of adding nerve roots and denticulate ligaments within this simpler modeling framework.

Figure S5 compares, at the time of flow reversal ($t/T = 0.7$), CFD predictions obtained using model (I), with and without microanatomy, against results obtained using model (V) with microanatomy. This comparison allows us to evaluate the relative impact of boundary-condition choice and microanatomical complexity on the instantaneous velocity fields and their agreement with PC-MRI measurements.

We then focus on how the inclusion of microanatomical features affects the anterior–posterior flow distribution when using the simplest boundary-condition model (I). Figure S6 compares the anterior and posterior flow-rate waveforms for the unobstructed canal (w/o) and for the canal including nerve roots and denticulate ligaments (N+L). The corresponding stroke volumes, computed by temporal integration of these flow-rate signals over one cardiac cycle, are reported in Table S5.

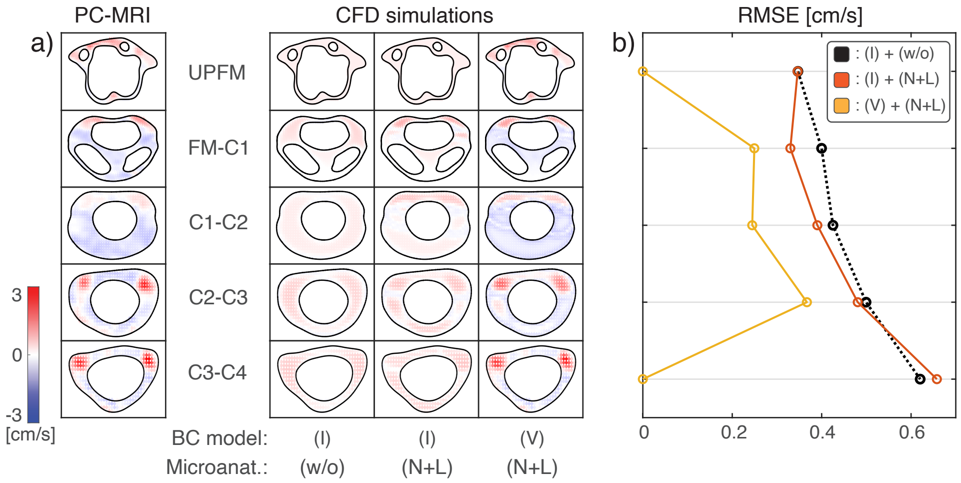

Fig. S5 Comparison of CFD flow predictions at the time of flow reversal ($t/T = 0.7$) for different boundary-condition models and microanatomical configurations. (a) CFD results correspond to boundary-condition model (I) without microanatomy (w/o), model (I) with nerve roots and denticulate ligaments (N+L), and model (V) with microanatomy (N+L). (b) Root-mean-square error (RMSE) between CFD and PC-MRI velocity fields for each configuration.

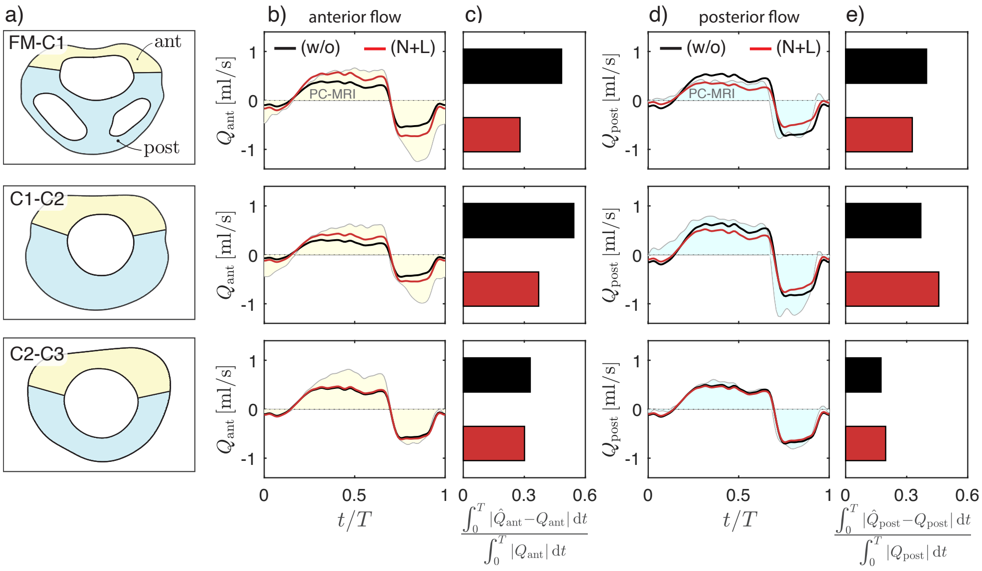

Fig. S6 Comparisons of CFD-predicted anterior–posterior flow distribution obtained using boundary-condition model (I), for the unobstructed canal (w/o, black) and for the canal including nerve roots and denticulate ligaments (N+L, red).

Table S5 Stroke volume (V_s) in the anterior and posterior regions, and their ratio, obtained by temporal integration of the anterior and posterior flow-rate signals shown in Fig. S6.

	Anterior V_s (ml)			Posterior V_s (ml)			Ant/Post		
	MRI	(w/o)	(N+L)	MRI	(w/o)	(N+L)	MRI	(w/o)	(N+L)
FM–C1	0.32	0.18	0.26	0.19	0.24	0.16	1.66	0.73	1.62
C1–C2	0.28	0.14	0.19	0.34	0.28	0.23	0.81	0.48	0.84
C3–C4	0.26	0.19	0.20	0.21	0.22	0.21	1.21	0.88	0.99

S5 PC-MRI uncertainty quantification

To assess the accuracy of the PC-MRI CSF velocity measurements, a pulsatile flow phantom experiment was conducted.

A programmable pulsatile pump (SuperPump, ViVitro Labs) was connected via rigid tubing to a custom-built phantom. The phantom represents the spinal SAS as the annular region between two eccentrically placed rigid cylinders. The inner cylinder radius was $R_i = 4.95\,\mathrm{mm}$ and the outer cylinder radius $R_o = 10.25\,\mathrm{mm}$, defining an average gap width $h = R_o - R_i = 5.3\,\mathrm{mm}$. The total length of the rigid section was $L = 30\,\mathrm{cm}$. The eccentricity, defined as the distance between the cylinder centers, was $e = 3\,\mathrm{mm}$. At the distal end, a compliant section was incorporated to accommodate the stroke volume imposed by the pump.

Water was used as the working fluid, with kinematic viscosity $\nu = 1\,\mathrm{cSt}$. The pump was operated at a frequency $f = 1\,\mathrm{Hz}$ (angular frequency $\omega = 2\pi\,\mathrm{s}^{-1}$). Based on the average gap width, the corresponding Womersley number, $Wo = h\sqrt{\omega/\nu}$, was $Wo = 12.4$, a value representative of CSF flow in the human spinal canal. A stroke volume of $V_s = 5.2\,\mathrm{ml}$ was prescribed.

Velocity measurements were acquired at the midpoint of the rigid section, 15 cm downstream of the inlet, in a plane perpendicular to the cylinder axes. MRI acquisition parameters were identical to those used for the in-vivo measurements (section 2.1 of the main manuscript), with a velocity encoding value of VENC = 15 cm/s. Data processing was performed in MATLAB following the methods described in section 2.2.2. Magnitude images were used to segment the region of interest corresponding to the annular flow domain, and phase images were converted to velocity in the through-plane (x-direction). The volumetric flow rate was obtained by spatial integration of the velocity field, $Q_{\mathrm{MRI}}(t) = \int_{\mathrm{ROI}} u_{\mathrm{MRI}}(t, y, z)\,\mathrm{d}y\,\mathrm{d}z$. Inspection of $Q_{\mathrm{MRI}}(t)$ revealed a periodic waveform that deviated slightly from a pure sinusoid (Fig. S7a). The measured flow rate signal was therefore decomposed into its first 20 Fourier harmonics.

The experimentally measured velocity fields were compared with numerical simulations performed in COMSOL Multiphysics. The model consisted of a two-dimensional cross-section of the eccentric annular domain, with geometry matching the experimental radius ratio and eccentricity. Assuming fully developed, unidirectional flow driven by a time-periodic longitudinal pressure gradient, the governing Navier–Stokes

equations reduce to the classical Womersley formulation,

$$\frac{\partial u}{\partial t} = P_\ell(t) + \frac{1}{Wo^2}\,\nabla_T^2 u, \tag{S3}$$

where $\nabla_T^2 = \partial^2/\partial y^2 + \partial^2/\partial z^2$ denotes the transverse Laplacian. Here, the pressure gradient is scaled by its characteristic amplitude $\Delta_\ell p$, velocity by $\Delta_\ell p/(\rho\omega)$, time by ω^{-1}, and length by the representative gap width h. The pressure gradient and the velocity were decomposed into Fourier modes as

$$P_\ell(t) = \mathrm{Re}\left\{\sum_{n=1}^{\infty} \mathrm{i}nP_n \mathrm{e}^{\mathrm{i}nt}\right\}, \qquad u(t,y,z) = \mathrm{Re}\left\{\sum_{n=1}^{\infty} u_n(y,z)\,\mathrm{e}^{\mathrm{i}nt}\right\}, \tag{S4}$$

yielding a sequence of boundary-value problems for each harmonic,

$$u_n + \frac{\mathrm{i}}{n\,\mathrm{Wo}^2}\,\nabla_T^2 u_n = P_n, \qquad n = 1, 2, \ldots, \tag{S5}$$

subject to no-slip boundary conditions, $u_n = 0$, on both cylinder walls bounding the annular domain.

Each harmonic problem (Eq. (S5)) was solved numerically using the finite element method in COMSOL. The Fourier coefficients P_n were chosen such that the resulting volumetric flow-rate waveform matched the experimentally measured (nearly sinusoidal) flow rate shown in Fig. S7a, retaining 20 modes—consistent with the harmonic content of the measured flow-rate waveform. After solving the boundary-value problems for all retained harmonics, the time-dependent velocity field was reconstructed by recomposition of the Fourier series given in Eq. (S4). The resulting velocity fields were then rescaled to recover dimensional velocities. This procedure enables a direct, quantitative comparison between the predictions and the experimentally measured PC–MRI velocity fields. The discrepancy between theory and experiment was quantified using the root-mean-square error (RMSE), as defined in section 2.5,

$$\mathrm{RMSE}(t) = \left\{\frac{1}{N_p}\sum_{j=1}^{N_p}\left[u_{\mathrm{MRI},j}(t) - u_j(t)\right]^2\right\}^{1/2}, \tag{S6}$$

where the summation is performed over all voxels within the annular region of interest, and N_p denotes the total number of voxels included in the comparison. For the flow conditions in the phantom ($Wo \approx 12$), the thickness of the oscillatory Stokes shear-wave layer is of the order of $\sqrt{\nu/\omega} \approx 0.4\,\mathrm{mm}$. This length scale is smaller than the in-plane voxel size of the PC–MRI acquisitions (0.625 mm), implying that the steep velocity gradients near the rigid walls cannot be fully resolved. As a result, a quantitative pointwise comparison between theory and experiment in the immediate vicinity of the boundaries is not meaningful. Accordingly, in the computation of the RMSE, the summation over pixels within the annular region excludes the two pixels adjacent to each solid boundary.

Figure S7b presents a side-by-side comparison of the velocity distributions measured by PC–MRI (left) and the corresponding numerical predictions (right). The PC–MRI measurements successfully capture the characteristic Womersley-type flow in the annular phantom. Consistent with the relatively large Womersley number, the oscillatory Stokes shear-wave layers are thin and confined to the immediate vicinity of the walls. Visual inspection indicates good overall agreement between measured and predicted velocity fields throughout the cardiac cycle. Quantitatively, the cycle-averaged RMSE is $\langle \mathrm{RMSE} \rangle = 0.33\,\mathrm{cm/s}$. When compared to the time-averaged mean velocity defined from the stroke volume as $v_\mathrm{c} = 2V_s(\omega/2\pi)/\left[\pi(R_o^2 - R_i^2)\right] \approx 4.1$ cm/s we obtained a normalized error of $\langle \mathrm{RMSE} \rangle / v_\mathrm{c} = 0.081$.

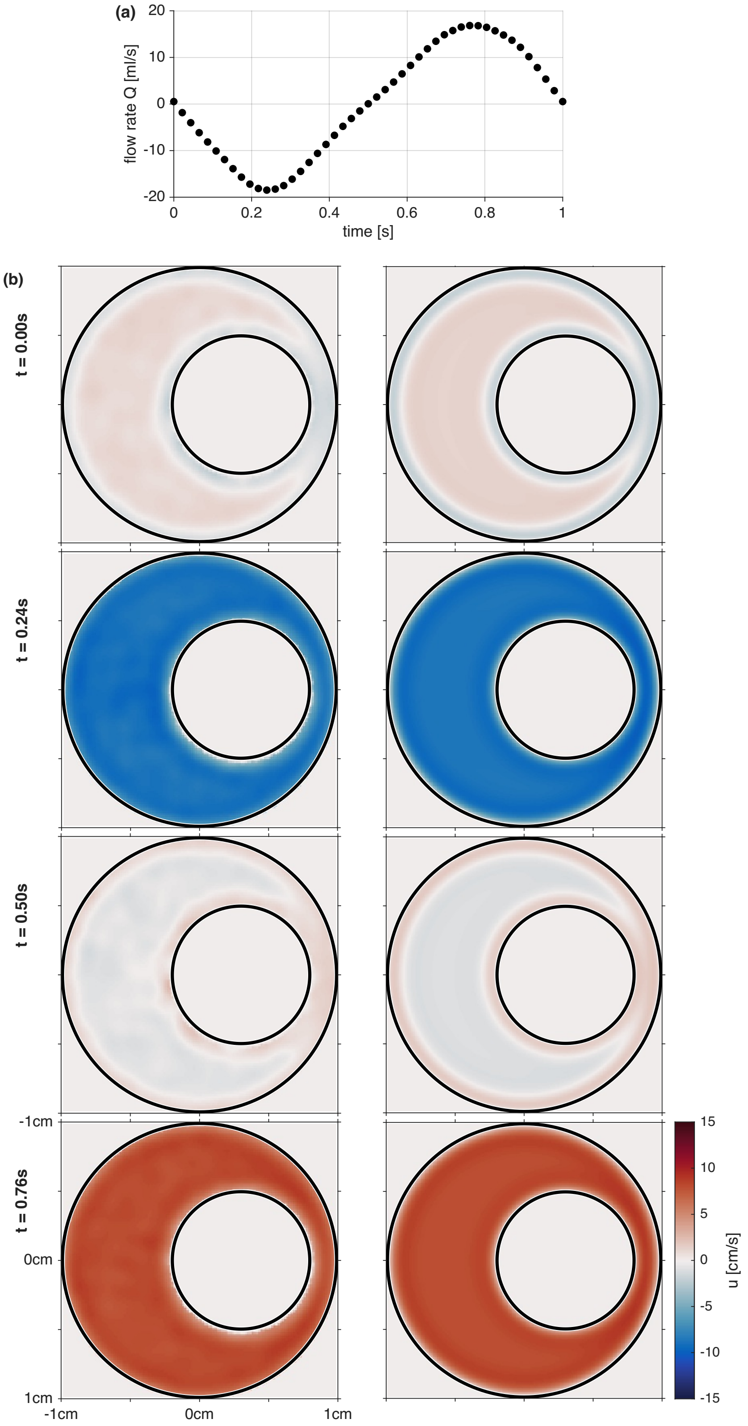

Fig. S7 (a) Volumetric flow rate imposed in the phantom by the pulsatile pump. (b) Velocity distributions in the phantom at four representative instants of the cardiac cycle; PC-MRI measurements (left) are compared with predictions obtained from the numerical model (right).
